# Predictive wave engineering in polymer phononic materials *via* viscoelastic–geometric coupling

**DOI:** 10.1039/d6mh00395h

**Published:** 2026-06-03

**Authors:** Sidharth Beniwal, Ranjita K. Bose, Anastasiia O. Krushynska

**Affiliations:** a Engineering and Technology Institute Groningen (ENTEG), Faculty of Science and Engineering, University of Groningen Groningen The Netherlands s.beniwal@rug.nl

## Abstract

Additive manufacturing has emerged as the most accessible option for fabricating polymer-based phononic materials, enabling complex architectures for advanced wave-control applications. However, improper or oversimplified material characterization often limits predictive accuracy and experimental reproducibility in wave control. Here, we establish an experimentally validated framework that integrates experimentally characterized viscoelastic material properties with systematic design variations to achieve accurate numerical predictions and experimental validation of wave dynamics in polymer phononic materials. We use the simplest disc-ligament designs of phononic crystals, analogous to mass-spring systems, and plot condensed band diagrams to examine the sensitivity of band gaps to systematic variations in unit-cell geometry and material distribution, including controlled porosity. Finite-element simulations incorporating experimentally measured viscoelastic properties are compared with transmission experiments across multiple geometries and polymer types, achieving close agreement between predicted and measured transmission responses. Overall, these findings provide a framework for accurate prediction of wave dynamics in additively manufactured polymer phononic materials.

## Abstract 2

Experimentally characterized viscoelastic properties combined with geometric design enable predictive wave engineering in polymer phononic materials, bridging the gap between numerical predictions and experimental observations.

## Graphical abstract

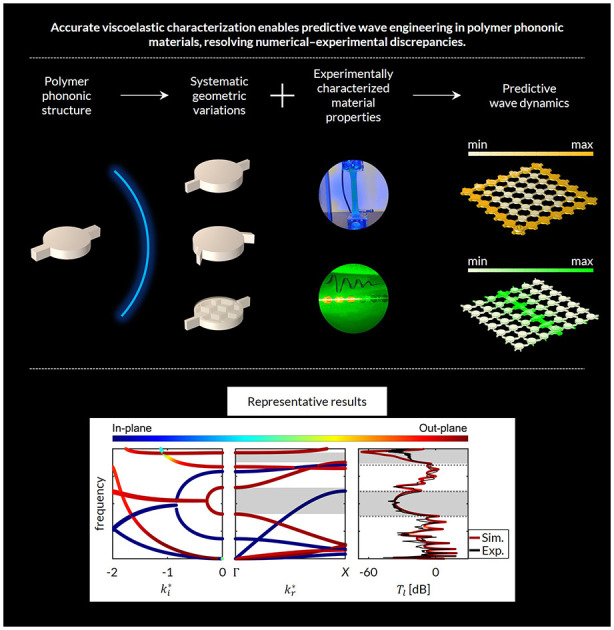

**New concepts**

Accurate prediction of wave propagation remains a major challenge in polymer phononics, with discrepancies between simulations and experiments often attributed to manufacturing defects. Here, we show that accurate material characterization is the key to resolving this mismatch. By integrating experimentally measured viscoelastic properties into finite-element models, we establish a predictive wave-engineering framework that accurately captures wave dynamics across different materials, manufacturing techniques, wave-control mechanisms, and environmental conditions. The framework also enables systematic tuning of wave propagation through geometric design and controlled porosity, shifting polymer phononics from trial-and-error development toward predictive engineering.

## 1 Introduction

The ability to manipulate elastic waves in phononic materials has created a fundamentally new basis for next generation solutions in vibration insulation,^1–3^ signal processing,^1–4^ frequency multiplexing and conversion^5–7^ energy harvesting,^2,8–10^ flow control,^11–13^ and many other applications. Most of such structures or their prototypes are manufactured from polymers,^14–17^ whose viscoelastic nature can complicate the relationship between a phononic geometry and its dynamic response, preventing reliable estimates of wave dynamics. These complications are even more critical for additively manufactured polymer phononics. Additive manufacturing is becoming the most accessible and scalable route for the fabrication of phononic materials, ensuring the precision and repeatability of intricate architectures,^16,17^ especially by using relatively inexpensive and simple techniques such as fused deposition modeling (FDM) and stereolithography (SLA).^15^ However, the viscoelastic properties of additively manufactured polymers are still insufficiently studied, limiting the use of polymer phononic structures in applications.

Despite substantial advances in the field of phononic materials, reliably predicting the dynamic characteristics of polymer-based architectures remains a challenge. The theoretical links between viscoelastic material behavior and wave dispersion have been established a while ago;^18–24^ however, these formulations have not been consistently translated into accurate predictions of experimentally observed responses. As a result, experimental and numerical results often mismatch, appearing as shifts in band gap frequencies,^25–27^ reduced attenuation magnitude,^27–35^ band gap broadening or narrowing,^26,31–37^ disappearance or blurring of predicted band gaps,^26,28–32,37,38^ unwanted drop in signal levels,^26,32,33,37^ and phase-differences in frequency response.^25,39^

These differences are often attributed to geometric or manufacturing imperfections. However, such explanations have not been thoroughly verified and do not fully account for observed mismatches. Although various viscoelastic models have been proposed to describe polymer behavior, these models are mainly *ad hoc* and must be manually tuned to match experimental data over limited frequency ranges. Related viscous parameters often lack physical meaning, are valid for limited frequencies, and cannot accurately describe the mechanical response of additively manufactured structures without prior calibration.^18,40^ As a result, numerical predictions often fail to reproduce measured dynamic characteristics, even if stiffness and damping are effectively frequency independent and viscous losses are minimal.^33,37,39^ This lack of predictive accuracy limits the transition of phononic structures to robust, application-ready devices. The need for a validated framework that enables accurate predictions from numerical models for any phononic structure is, thus, crucial to precise wave engineering in polymer and, especially, additively manufactured designs.

Wave manipulation in phononic materials is primarily governed by three mechanisms: Bragg scattering, arising from wave interference in periodic lattices;^41^ local resonance, where constituent elements vibrate independently at pre-defined frequencies;^42^ and inertial amplification, which enhances mechanical momenta by activating localized rotations.^43^ Reduced models that account for effective stiffness, mass, impedance, and damping can approximate the structural dynamics driven by these mechanisms. In additively manufactured polymer structures, variations in layer adhesion, porosity, and other 3D-printing parameters introduce time (frequency) dependencies in effective dynamic characteristics. When the mechanical behavior of constituent polymers is not properly captured, approximations fail to predict structural dynamics accurately, and numerical simulations yield erroneous estimates. Then, the calculated dynamic characteristics differ from the experimental data, with band gaps and transmission peaks appearing shifted, narrowed, or absent. Even if viscous losses are small, the mismatch persists because the models do not fully capture the true behavior of the constituent additively manufactured polymer.

Here, we develop and experimentally validate an approach to reliably predict wave dynamics in additively manufactured polymer phononic materials. The approach couples viscoelastic modeling with systematic modifications to a phononic geometry. We exemplify the workflow by considering phononic designs composed of circular disks connected by thin ligaments, fabricated from widely used, commercially available thermoplastic polymers – poly lactic acid (PLA) and Acrylonitrile–Butadiene–Styrene (ABS) – and by manufacturing test samples on FDM hobbyist-type 3D printers. The mechanical properties obtained from characterization of the 3D-printed polymers are incorporated into a linear-viscoelastic finite-element model (FEM), which is validated through non-contact pitch-catch transmission experiments. This experimental-numerical framework is applied to study the evolution of classical wave-control mechanisms in polymer phononic structures with non-zero viscosity. We also propose two design strategies for fine-tuning bandgap frequencies through controlled structural porosity and pluripotent inertial amplification. We prove that inevitable manufacturing imperfections have a negligible influence on wave propagation, contrary to common belief, whereas small volumetric or geometric changes can produce measurable effects on wave dispersion and transmission characteristics. Finally, we demonstrate that the proposed approach can be used to accurately predict wave characteristics in essentially three-dimensional complex phononic structures manufactured with a higher resolution, *e.g.*, using stereolithography (SLA), confirming its universality for additively manufactured phononics, in general. Therefore, our results resolve long-standing mismatch between numerical predictions and experiments for polymer phononic structures by bridging material characterization and structural dynamics through experimentally informed numerical predictions.

## 2 Design philosophy of phononics with traditional wave control mechanisms

**Fig. 1:**
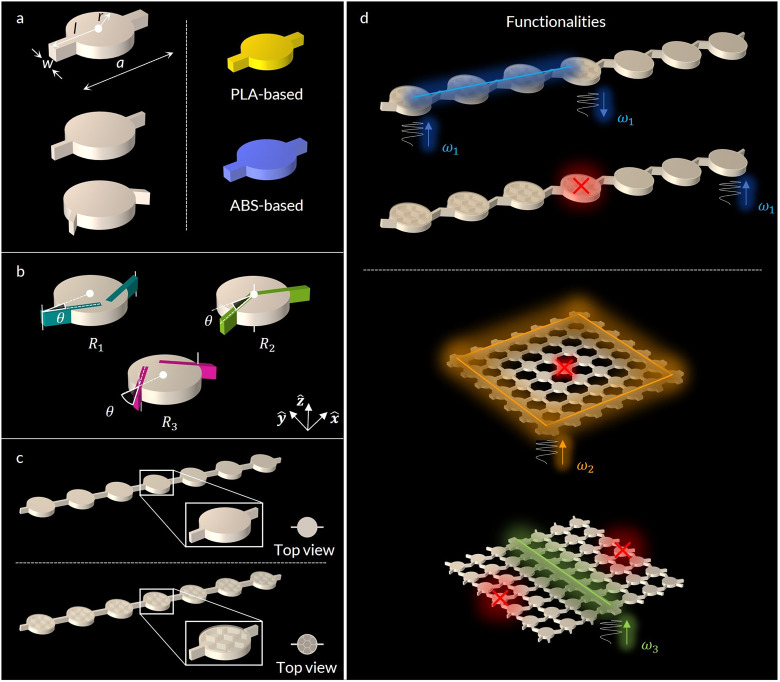
**Design approach and possible functionalities for phononic strips and plates with solid and porous parts.** (a) Phononic unit cells with thick, thin, and rotated ligaments for activating classical wave manipulation mechanisms: Bragg scattering, local resonance, and inertial amplification, respectively. Each design is fabricated here using two commonly used polymers: PLA or ABS. (b) Different approaches, *R*_1_, *R*_2_, and *R*_3_, to implement the inertial amplification mechanism by in-plane rotation of thin ligaments, with a constant volume of a unit cell. (c) Phononic strips with solid (0% porosity) and porous (5–42.5% porosity) unit cells, where porosity is introduced by honeycomb infill. (d) One-way wave propagation (acoustic diode) and waveguiding can be achieved by combining solid and porous unit cells and/or unit cells with different wave manipulation mechanisms.

We analyze the effects of stiffness, inertia, and geometry on wave propagation in phononic media by considering a simple yet practically relevant phononic configuration: a periodic array of circular disks connected by straight rectangular ligaments. Such continuum analogs of periodic mass-spring systems can be readily used to implement the three mentioned wave control mechanisms.^3,44–47^ Thick straight ligaments and small disks can open Bragg band gaps;^41,48^ thin ligaments and large disks enable the activation of locally resonant band gaps (Fig. 1a).^42^ If the ligaments are inclined, the inertial amplification mechanism is activated.^43^ Note that ligaments can be inclined in different ways, *i.e.*, the inclination angle *θ* can be defined in various ways, as shown, *e.g.*, in Fig. 1b. Differences in wave dynamics caused by the inclination of the ligaments are discussed in Section 2.1.

The described designs can exhibit different frequency ranges of wave attenuation depending on the constituent material. Here, we first consider phononic structures made of either biodegradable polylactic acid (PLA) or organic copolymer acrylonitrile butadiene styrene (ABS). These are the two most widely used thermoplastics for cost-effective prototyping of additively manufactured phononic structures.^16,40,49–57^ We assume a constant out-of-plane thickness *h* = 4 mm and a fixed volume of material *V* = 1344 mm^3^ in each unit cell. We also fix the center-to-center distance *a* = 32 mm for configurations with straight (non-inclined) ligaments.

**Fig. 2:**
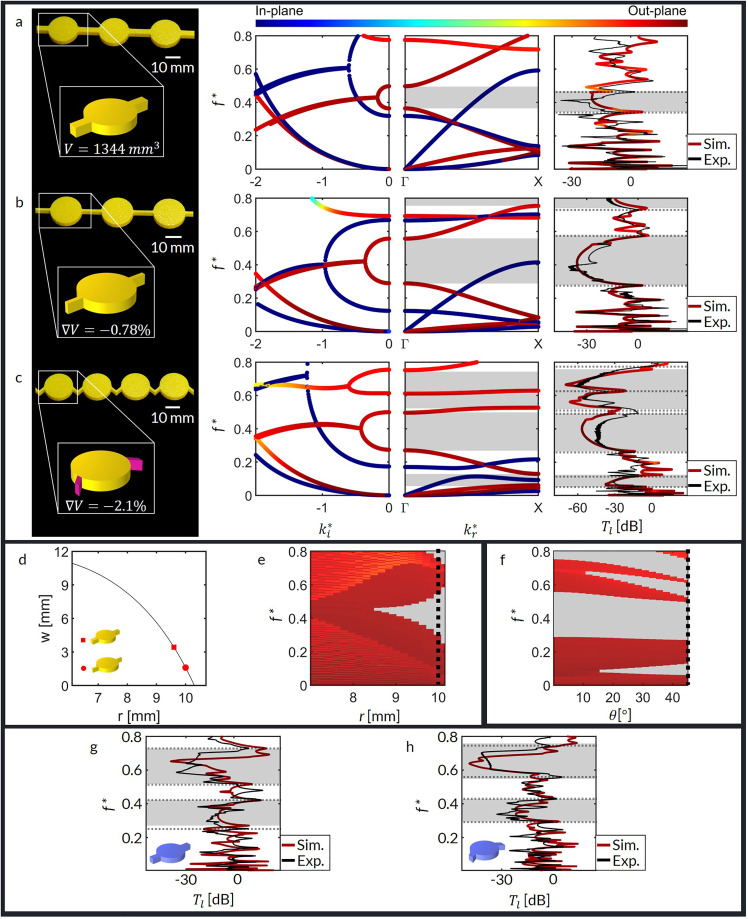
**Dynamic properties of phononic strips with solid parts.** (a)–(c) Sections of PLA strips together with their dispersion and transmission curves for the following unit cell designs: (a) straight thick ligament (disc radius *r* = 9.6 mm, ligament width *w* = 3.6 mm, unit cell size *a* = 32 mm), (b) straight thin ligament (*r* = 10 mm, *w* = 1.6 mm, *a* = 32 mm), and (c) rotated thin ligament (*r* = 10 mm, *w* = 1.6 mm, ligament length *l* = 16 mm, ligament rotation *θ* = 45°, and *a* = l (1 + cos *θ*) − *w* sin *θ* = 26.18 mm). (d) *w* as a function of *r* for fixed *a* and unit-cell volume. (e) Condensed band structure diagram for straight-ligament strips as a function of *r*. (f) Condensed band structure diagram for rotated thin-ligament strips as a function of *θ*. Black dotted lines in (e) and (f) indicate the parameters used in (b) and (c), respectively. (g)–(i) Numerical and experimental transmission results for three designs of ABS strips: (g) straight thick ligament (*r* = 11.45 mm, *w* = 4 mm, *a* = 33 mm), and (h) rotated thick ligament (*r* = 11.45 mm, *w* = 4 mm, *θ* = 32°, *a* = l (1 − (*θ*/45[deg]) × 0.11) = 30.34 mm). The normalized frequency f* = *fa*/*c*_s_ and wavenumber k* = *ka*/π are given for bands with a strong out-of-plane polarization (*P* > 0.75). The numerical band gaps are shaded in gray, while the dotted gray lines indicate the bounds of the experimental band gaps.

The calculated band structure diagrams of the three (reference) configurations, with the corresponding unit cells shown in Fig. 1a, are given in Fig. 2a–c (see Section 5.4.2 for calculation details). We use normalized frequencies f* = *fa*/*c*_s_ and wave numbers k* = 2π/*a*, where *f* is the frequency in Hz and 
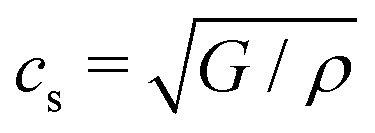
 is the shear wave velocity in a bulk material with shear modulus *G* and material density *ρ*.

The band structure diagram in Fig. 2a has a single narrow band gap for out-of-plane modes centered on f* ≈ 0.42. This band gap is Bragg-type, as indicated by the parabolic shape of the imaginary branches peaking at the mid-gap frequency. The unit cell analyzed has the radius of the disk *r* = 9.6 mm, the length of the ligaments *l* = 16 mm, and the in-plane thickness *w* = 3.6 mm. The width of the band gap can be changed by redistributing the material between the disk and the ligaments. For example, for *r* = 10 mm, *w* = 1.6 mm, and *l* = 16 mm, the band gap increases, and a second band gap emerges around f* ≈ 0.775 (Fig. 2b). The flattened bounds of the two band gaps indicate the activation of local resonance modes,^1,42,58^ although the imaginary branches preserve their parabolic shape of an increased magnitude (*cf.*Fig. 2a and b), signifying stronger wave attenuation.^1,58–60^

The constraint imposed on the fixed volume of material and the out-of-plane thickness *h* provides a relationship between in-plane parameters of the unit cell, π*r*^2^ + 2(*l* − *r*)*w* = const, shown in Fig. 2d. Here, the red markers correspond to the configurations shown in the diagrams in Fig. 2a and b. The condensed band structure diagram for the radius values described by this relation (Fig. 2e) shows the opening of the lowest band gap for *r* ≈ 8.48 mm. The width of this band gap increases with increasing *r*, while the mid-frequency of the central band gap decreases from f* ≈ 0.45 to f* ≈ 0.41. The widest band gap is obtained for the thinnest ligaments and is attributed to the simultaneous activation of the Bragg and local-resonance mechanisms due to the largest mass-stiffness disparity.^1,3,61^

The width of the lowest band gap can be further increased by activating inertial amplification by inclining the ligaments. For example, the ligaments can be rotated about the central axis *ẑ* of the unit cell by angle *θ* to a maximum value of *θ* = 45° (design *R*_2_ in Fig. 1b). To maintain a constant volume of material, the size of the unit cell should be reduced to *a* = l (1 + cos *θ*) − *w* sin *θ*. The faces of the ligaments at the boundaries of the unit cell are retained flat to allow the application of periodic Floquet–Bloch boundary conditions. The band structure diagram for the unit cell with *θ* = 45° (Fig. 2c) has an extra low-frequency band gap centered around f* = 0.1, suggesting advanced low-frequency wave attenuation. The condensed band diagram for various rotation angles (Fig. 2f) shows the evolution of the width and frequencies of the band gaps with increasing *θ*. New band gaps are opened at f* ≈ 0.1 and above f* ≈ 0.64 for *θ* ≠ 0. As the rotation angle increases, these band gaps widen, decreasing the frequency spacing between localized modes. In contrast, the Bragg band gap, centered at f* ≈ 0.42, maintains its almost constant width across all inclination angles. The lower bound of the lowest band gaps decreases with increasing rotation angle, which corresponds to improved low-frequency wave attenuation desirable in applications.^3,62–65^ This occurs because the effective inertia of the rotated ligaments is increased, slowing the phase velocity of the propagating waves.

To validate these findings, we performed transmission tests on finite-sized samples consisting of 7 unit cells. Note that this length is sufficient to adequately represent the wave dynamics of periodic phononic structures, without noticeable boundary and finite-size effects (see Section S2.4 and Fig. S8 for details.) We have calculated and measured the magnitude of transmitted out-of-plane waves at the surface of the 5th unit cell from the excitation point for each FDM sample 3D-printed from PLA (Section 5.3). The numerical (red) and experimental (black) transmission curves in Fig. 2a–c indicate clear band gaps (gray shaded) identified as frequency regions with transmission drops exceeding 20 dB.

The numerical curves predict the band gaps at frequencies similar to those in the dispersion analysis, for all analyzed configurations. The Bragg band gap, centered at f* = 0.42, is more pronounced for the configuration with the thin and rotated ligaments rather than for the thick ligament structure, in agreement with the larger magnitude of the imaginary branches compared to that for the thick-ligament design (Fig. 2a–c). The structure with inclined ligaments exhibits strong wave attenuation at lower frequencies, around f* = 0.1, and a particularly deep transmission dip near f* = 0.64, making it attractive for wave-attenuation applications.

The excellent agreement between the numerical and measured data was achieved by using experimentally derived values of the material mass density and frequency-dependent stiffness in the numerical simulations. A quantitative comparison between the experimental and numerical data is provided in Table S2.

We emphasize that small deviations in the mass density cause shifts in the computed band-gap frequencies. Specifically, for our geometries, a density underestimate of 5% results in an upward shift of dispersion branches of up to 3–3.5%, in agreement with ref. 66. Similar, though less pronounced, effects are observed for stiffness moduli. For example, a 5% underestimate of *E*′, *G*′, and *G*″ results in a decrease in the mid-frequency of the band gap by 0.6%, 1.6%, and 2.9%. The combined effect of underestimating all parameters by 5% is a downward shift of the mid-gap frequency by 2.55%. Note that we consider here the mass density and elastic moduli as homogeneous, whereas FDM-printed polymers are inherently heterogeneous and anisotropic. The effects of print orientation on these characteristics are discussed in Section S1. Despite measured deviations of up to 30% in directional elastic moduli, wave dispersion and transmission simulations based on anisotropic material properties differ slightly from those based on the isotropic model and only at higher frequencies, above the first bandgap (see Section S2.1). Therefore, for simplicity, we continue assuming an isotropic response for 3D-printed PLA and ABS.

Surprisingly, if one neglects material viscosity and considers a pure elastic case, the downward shift in the mid-gap frequency is 31.32%. We argue that such a shift is a general trend across phononic systems, independent of a specific geometry. To confirm this, we have estimated the mid-frequency of the lowest band gaps for the syndiotactic and isotactic phononic structures proposed in ref. 39 and reproduced by us in PLA (see Section S3.2 for details). For both configurations, neglecting viscosity has resulted in the frequency shifts of −31.51% (isotactic) and −31.02% (syndiotactic), as shown in Fig. S12. These results confirm the leading role of the constituent material (PLA) rather than geometric dependence and emphasize that the accurate characterization of the material is crucial for reliable numerical modeling of wave dynamics in phononic structures (see Section 4 for more details).

We have also estimated the wave transmission in geometrically identical additively manufactured phononic structures made of ABS; see Fig. 2g and h. The experimental data reveal an excellent agreement with the numerical predictions. For both PLA and ABS, we assume perfect bonding and continuity in the printed structures, neglecting interlayer interfaces created by FDM and thus the presence of weak planes. This is a valid assumption at comparately low analyzed frequencies (below 18 kHz) since the characteristic size of interfacial defects is comparable to the layer thickness (0.4 mm for the used FDM 3D-printing parameters; see Section 4), while the minimum analyzed wavelength of shear waves in bulk PLA or ABS equals 35.5 mm. This two-order-of-magnitude difference suggests that propagating waves are insensitive to small manufacturing irregularities. To confirm this further, we manufactured the same disk-ligament configurations from hard resin using stereolithography (SLA), which delivers at least one order higher printing resolution, perfect layer bonding, and isotropic material behavior, and again found a very good agreement between our predictions and experimental results (see Section S4.3, Fig. S16 and Table S3 for quantitative comparison).

Altogether, these results further confirm that accurate estimates of the mechanical properties and mass density of 3D printed polymers, including (small) viscoelastic losses (see Section 4), are essential for reliable prediction of wave transmission and frequency band gaps in phononic structures, regardless of a constituent polymer, manufacturing technique, or geometry-driven wave control mechanisms.

Based on this knowledge, we propose two distinct approaches to fine-tuning wave dynamics in phononic structures as explained below.

### 2.1 Pluripotent designs of inertial amplification phononics

It is well known that in mass-spring phononic systems and their continuous counterparts, the inertial amplification mechanism can be activated by using rotational springs and inclined thin elements, respectively.^3,43–45,62,63,67^ In our designs, this mechanism can also be activated by rotating the ligaments, for example, about the *ẑ*-axis in the center of the unit cell (*R*_2_ in Fig. 1b). This is not the only possibility, since ligaments can rotate about various axes, each resulting in potentially distinct wave-dynamics characteristics.

Here, we analyze three approaches to choosing the rotation axis *ẑ*. Specifically, we consider ligaments rotated about (1) an out-of-plane axis centered at the edge of the ligament (*R*_1_ in Fig. 1b); (2) an axis in the center of the disk (*R*_2_ in Fig. 1b, Sections S2.1), and (3) an axis at the intersection of a ligament with the edge of the disk (*R*_3_ in Fig. 1b). We fix the width of the ligaments to 1.6 mm and the radius of the disk to 10 mm and calculate the condensed band structure diagrams for these three scenarios (Fig. S11a–c). The calculation results are reported in terms of normalized frequency f* = *fa*/*c*_s_, as the size of the unit cell for each configuration varies.

As the rotation angle increases, the band gaps in all cases shift toward lower frequencies, as can be expected for the inertial amplification mechanism. However, several differences are observed due to the choice of rotation angle. First, *R*_2_ designs reveal the strongest inertial amplification effect, as they have the widest band gaps among the three cases that are substantially shifted toward lower frequencies as the rotation angle increases (Fig. S11b). Since *a* = 2*l* cos *θ* − *w* sin *θ*, this design has the smallest unit cell for a fixed rotation angle (Fig. S11 and Section S3.1).

Among the other two designs, *R*_1_ has the most distinct dynamic behavior: the width of the band gaps decreases with increasing rotation angle (Fig. S11a), which contradicts common expectations for inertial amplified wave dynamics.^3,43,45,62,63,67^ In addition, the lowest band gaps around f* = 0.1 and f* = 0.6 in the *R*_1_ designs open at much larger angles than in *R*_3_ (Fig. S3), and the band gap around f* = 0.8 completely closes at *θ* = 27.5°, while it widens in *R*_3_ with increasing *θ*. The *R*_1_ designs have fewer band gaps while the *R*_3_ designs have wider band gaps at higher frequencies. The unit cell sizes for *R*_1_ and *R*_3_ are estimated from the relations *a* = 2*l* − *w* sin *θ* and *a* = l (1 + cos *θ*) − *w* sin *θ*, respectively.

These findings demonstrate that the choice of the rotation axis influences the wave propagation characteristics governed by the inertial amplification. The explanations for this can be found in the modified geometric parameters and underlying physics. For example, the *R*_1_ configurations have the largest distances between the center of the unit cell and its boundary, 15.64 mm for *θ* = 22.5°, resulting in the maximum mass leverage that promotes the strongest inertial amplification. However, it also leads to the largest unit cell size, compensating for the gain by higher frequencies of the Bragg-type band gaps. Moreover, the effective mass–spring interactions at lower frequencies are reduced, and hence the width of the band gap is not optimal. In contrast, balanced performance can be achieved in *R*_3_ with a moderate unit cell size of 30.17 mm for *θ* = 22.5° combined with the smallest distance between the center of the unit cell and its boundary 15.38 mm. The widest band gaps are found in the *R*_2_ design with a moderate center-edge distance, 15.61 mm for *θ* = 22.5°, preserving a compact unit cell size of 28.84 mm. This enables the shift of the band gaps to lower frequencies and thus effective inertial amplification. The *R*_2_ configuration also leads to the appearance of asymmetry at lower rotation angles and mode coupling, allowing the opening of band gaps at lower rotation angles.

### 2.2 Controlled structural porosity for band gap fine-tuning

Our results show that variations in the unit cell geometry change both the effective stiffness and inertia, which, in turn, strongly influence wave propagation in a phononic structure. This suggests an alternative approach to modify these two characteristics and thus manipulate the dynamic characteristics – *controllable internal porosity*. It implies replacing the solid interior of a unit cell with a cellular infill of a predefined topology (*e.g.*, a honeycomb) while preserving external bounding walls. (Fig. 1c). The in-plane thickness of the honeycomb infill, *t*, can be varied to tune the level of porosity.

Here, we focus on the internal porosity of the disks because analysis of the ligaments with porous infills has revealed no significant effects on the wave dynamics, as expected given the already small thickness of the ligaments. The ligaments are thus modeled as fully solid in the remainder of this study. Note that the honeycomb lattice is surrounded by a thin wall of thickness 0.8 mm at the edges of the disk to prevent open pores and increase the mechanical integrity of the phononic structure.

We first analyze the thin-ligament phononic design (Fig. 2b) using disks with the highest porosity, 42.5%, which is permissible for the honeycomb infill given the minimum 3D-printable wall thickness of our 3D printer (see Section 5.1). Note that the same geometry with an empty interior (shell-type designs; see Section S2.5 and Fig. S9) can reach a porosity of up to 53.4%, which is a maximum value for this geometry. Importantly, this value is not restrictive for the proposed analysis and can, in principle, be increased further using other manufacturing techniques, subject to application-driven and fabrication constraints.

**Fig. 3:**
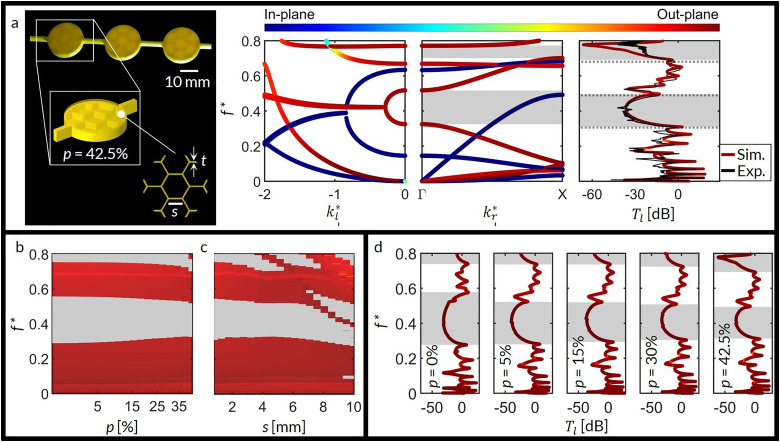
**Influence of controlled porosity on wave dynamics.** (a) Representative unit cell for 42.5% porosity, along with its dispersion curves and transmission spectra (both numerical and experimental). (b) and (c) Condensed band structure diagrams showing the effect of (b) porosity *p* and (c) honeycomb side length *s*. (d) Numerically calculated transmission curves for different porosity levels, demonstrating the change in band gap behaviour.

The dispersion and transmission curves for the porous phononic structure (Fig. 3a) have a qualitatively similar behavior to those for the solid counterpart (*cf.*Fig. 2b). The calculations show that the porous design has a narrower first band gap, while the second band gap opens at lower frequencies. The experimental transmission data (the black curve) confirm these observations. This change in wave dynamics is driven by a lower mass of the porous disk, which reduces the contribution of local resonance modes and thus decreases the band-gap width. Simultaneously, the reduced stiffness of the porous disks (as corroborated by quasi-static tensile tests; see Fig. 6) lowers the frequencies of higher-order localized modes, shifting the second band gap to lower frequencies while preserving a similar attenuation level within the band gap.

This reasoning is further confirmed by the variation of the band gap width with porosity *p* in the condensed band structure diagram, Fig. 3b. Clearly, small porosity (5%) introduces no noticeable modifications in band gap frequencies, while a complete band structure diagram (not shown) indicates an enhanced mode separation near f* = 0.52, also visible in the transmission curve in Fig. 3d. An increase in porosity shifts the band gaps to lower frequencies and decreases their widths. Similar effects are found by varying the side length of the infill honeycomb *s* for the configuration with maximum porosity for the honeycomb infill (42.5%), as shown in Fig. 3c. These findings demonstrate that both the location and the extent of the band gaps are sensitive to the geometric parameters of the infill pattern.

One could expect a stronger influence of porosity on the band gaps activated by the inertial amplification mechanism. Indeed, Figs. S11d–f show that the lowest band gaps in the highly porous configurations (42.5%) are opened at lower rotation angles compared to their solid counterparts (Fig. S11a–c), and the band-gap frequencies are shifted downward due to reduced effective stiffness (Fig. 6a and b).

These findings demonstrate that intentionally introduced internal porosity, as a design variable, enables control over the location and width of frequency band gaps, which can be used to fine-tune wave propagation characteristics in phononic structures. Finally, we note that the analysis aimed at decoupling stiffness and damping effects caused by porosity is beyond the scope of this study.

## 3 Enabling polymer phononic applications

The design strategies discussed above provide distinct ways of controlling wave propagation. We now combine them to realize dynamic behavior that does not arise in the individual configurations when considered separately. Specifically, we show that combining the design strategies enables time-invariant passive devices with directional wave filtering and waveguiding.

### 3.1 Quasi-1D multi-morphology polymer phononic chain

**Fig. 4:**
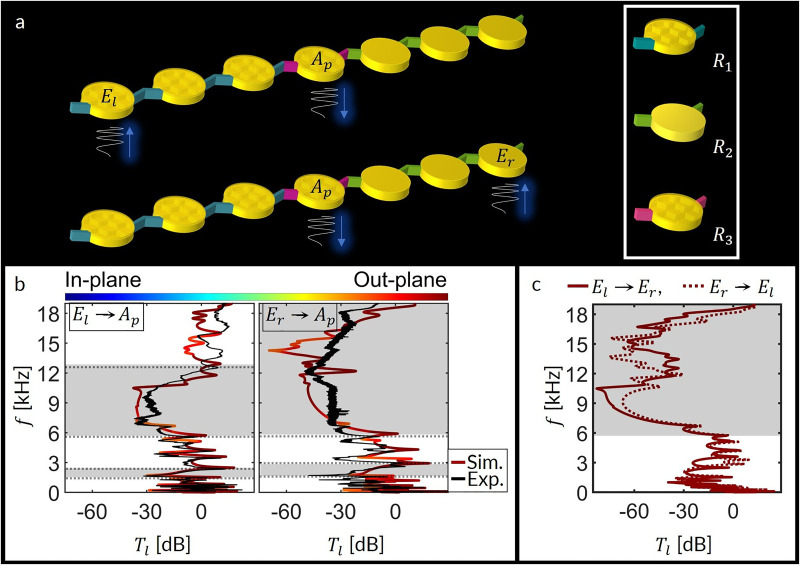
**Quasi-1D multi-morphology phononic chain.** (a) Conceptual layout of the structure designed to demonstrate the combined effects of pluripotent inertial amplification and controlled structural porosity. Discs 1–3 (left-to-right) follow the *R*_1_ configuration, disc 4 adopts the *R*_2_ configuration, and discs 5–7 are inspired by the *R*_3_ configuration. (b) Numerical and experimental wave transmission results when the structure is excited separately at points *E*_l_ and *E*_r_, with signal acquisition at point *A*_p_. (c) Total numerical transmission through the structure when excited separately at points *E*_*l*_ and *E*_*r*l_.

First, we consider a quasi-1D multi-morphology chain (Fig. 4a). Here, we use the term morphology in the material-science sense, referring to the structural form of material,^68^ and extend it to phononic structures. Specifically, we combine distinct unit-cell designs to form a single phononic chain, which we refer to as a multi-morphology phononic chain. Our exemplary configuration comprises inertial-amplification unit cells of different morphologies with controlled internal porosity, and it features spatial variations in the chain to enable direction-dependent wave transmission.

In the literature, distinct morphologies of phononic chains are typically obtained by varying the cross-section of a unit cell, *e.g.*, by alternating thick and thin beams, to create two topologically protected interface modes.^69–71^ In contrast, we generate distinct morphologies using pluripotent inertial-amplification unit cells and treat controlled internal porosity as an independent structural parameter. This enriches structural diversity and enables enhanced wave manipulation without relying solely on cross-sectional geometric variations.

We have first identified promising unit-cell designs for a multi-morphology configuration based on the condensed band structure diagrams in Fig. S11. For this, we determined overlapping frequency ranges with opposite dynamic responses (pass *vs.* stop bands). Based on this, the sequence {*R*_1_-porous, *R*_3_-porous, *R*_2_-solid}, *e.g.*, can be suitable to achieve direction-dependent transmission. In particular, the first three cells have an *R*_1_ design with 42.5% porosity; the middle cell has an *R*_3_ design with the same porosity; and the other three cells have a solid *R*_2_ design. All ligaments have an in-plane thickness 1.6 mm and are rotated by 22.5° to ensure connectivity between discs of radius *r* = 10 mm. The resulting structure is shown in Fig. 4a. Note that the number of unit cells with designs *R*_1_ and *R*_2_ can be larger; here, it is limited to three to ensure manufacturability, which is constrained by the print bed dimensions.

The experimental transmission data shown in Fig. 4b for the described multi-morphology FDM-printed PLA phononic chain reveal a clear directional dependence for waves propagating from the two structural ends to central point *A*_p_. Specifically, a signal excited at the point *E*_r_ experiences a broad attenuation of more than 25 dB between 5.7 kHz and 18 kHz when reaching point *A*_p_. In contrast, a signal excited at the point *E*_l_ is attenuated only from 5.6 kHz to 12.5 kHz. The numerical predictions are consistent with the experimental measurements. We note that this design exhibits reciprocal behavior, as the total transmission between *E*_l_ and *E*_r_ is identical in both directions, see Fig. 4c. Nevertheless, wave propagation depends on the location of the excitation point, reflecting the direction-dependent response imposed by spatial differences in the unit-cell morphology. Therefore, this multi-morphology phononic chain acts as a passive directional filter that can potentially be used in direction-aware sensing to identify, *e.g.*, the source of disturbances in safety monitoring, soft robotics, or other systems.

Other reported quasi-1D multi-morphology designs were realized in either lossless materials, such as metals,^69^ or lossy materials, such as silica.^70^ Metal structures show band gaps from 0.6 to 0.96 in normalized frequency (with respect to the velocity of shear waves in the respective bulk material), along with a narrow low-frequency band gap at 0.24 and a band-gap width of 0.05. Silica-based chains operate at much higher normalized frequencies and exhibit clear shifts between the experimental and numerical band gaps across the entire band structure diagram. Our PLA multi-morphology phononic chain supports broader band gaps that start at much lower normalized frequencies (0.25–0.5 for excitation at *E*_l_; 0.25–0.8 for *E*_r_) and reveal excellent agreement between experimental data and numerical predictions.

Therefore, we have experimentally demonstrated spatially dependent wave transmission in lossy 3D-printed polymers with a high predictive accuracy. Our multi-morphology design achieves broad, tunable band gaps in a lightweight polymer lattice, providing a modular 1D waveguide with precise control of elastic waves.

### 3.2 Waveguiding in multi-morphology polymer phononic plates

The multi-morphology designs can be realized in phononic plates, *e.g.*, to implement waveguiding, *i.e.*, to confine and direct elastic energy along a prescribed route. This can be done, *e.g.*, by engineering a “defect” path in a periodic lattice.

Defect paths are usually created in two ways. The first approach relies on selectively modifying the geometry or mechanical properties of a lattice or on removing unit cells to form localized transmission channels at band-gap frequencies.^72,73^ Such designs are often reported to be sensitive to fabrication tolerances, including geometric imperfections and misalignments. The second approach implements topological architectures that support wave propagation across domain interfaces with differing topological orders.^73,74^ The interfacial edge modes are protected by topology, allowing waves to propagate with negligible backscattering even in the presence of local defects or sharp bends. Here, we focus on the first approach to prove that apparent discrepancies between simulations and experiments in defect-based waveguiding predominantly arise from inaccurate material characterization rather than fabrication errors, as often assumed.

**Fig. 5:**
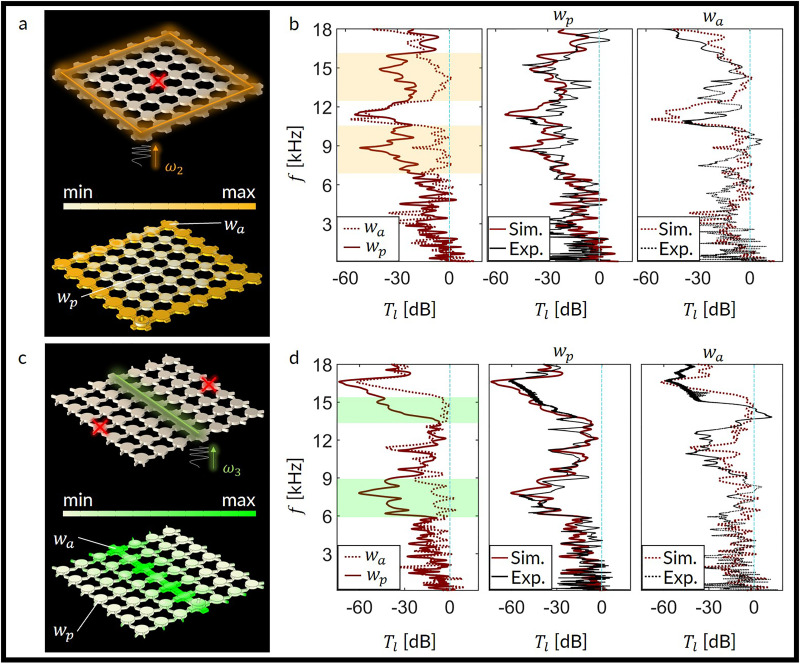
**Quasi-2D waveguide structures and their transmission characteristics.** (a) Edge-guided waveguide and corresponding displacement field. (b) Transmission characteristics of the edge-guided structure: numerical comparison of responses at *w*_*p*_ and *w*_*a*_ (left), and numerical–experimental comparison at *w*_*p*_ (middle) and *w*_*a*_ (right). (c) Central-guided waveguide and corresponding displacement field. (d) Transmission characteristics of the central-guided structure: numerical comparison of responses at *w*_*p*_ and *w*_*a*_ (left), and numerical–experimental comparison at *w*_*p*_ (middle) and *w*_*a*_ (right). The cyan dashed line indicates the 0 dB transmission level.

We analyze two scenarios: edge-waveguiding, where the wave propagation is confined to structural edges, and central-waveguiding, where wave energy propagates along a central part of the structure. Fig. 5a and c demonstrate the plate-type phononic configurations that implement these scenarios together with numerically estimated out-of-plane displacements at waveguiding frequencies. In both cases, periodic structural parts are formed by disks of *r* = 9.6 mm connected by thin straight or inclined (*θ* = 24°, *R*_2_ design) ligaments of width *w* = 2 mm, respectively. The defect paths are formed by stiffer unit cells: those with straight ligaments of in-plane thickness *w* = 8 mm connecting disks of radius *r* = 12 mm, for the edge-waveguiding configuration, and those with straight ligaments of width *w* = 10 mm connecting disks of radius *r* = 9.6 mm, for the central-waveguiding configuration.

The numerical and experimental transmission curves for the described structures are shown in Fig. 5b and d. The first (left-hand-side) plot depicts numerical results estimated at probes *w*_a_ (on the defect paths) and *w*_p_ (in the periodic lattice). The other two plots show the experimental *vs.* numerical transmission at and outside the defect paths, respectively. The wave transmission along the waveguiding path is nearly three orders of magnitude larger than that in the internal lattice, even though the side probe is positioned only three unit cells away from the defect path. This behavior confirms strong mode localization and indicates that waveguiding can be realized predictably in polymer phononic plates.

The overall performance of the plate-type phononic designs (Fig. 5a and c) in terms of guiding bandwidth, transmission contrast, mode localization, and robustness to imperfections is analyzed. Both configurations exhibit a transmission contrast of at least 25 dB between the defect and lattice propagation paths, demonstrating efficient energy confinement and strong mode localization. This contrast exceeds those reported for polymer-based phononic waveguides and is achieved without introducing topological or multi-material complexity.^75^ The waveguiding occurs in two frequency windows of approximately 3 kHz each, indicating moderate bandwidth; see highlighted regions in Fig. 5b and d). Although broadband waveguiding has been demonstrated in three-dimensional polymer structures,^3^ for plate-like lattices, it has only been achieved in metallic systems,^27,75,76^ to the best of our knowledge.

The close correspondence between the experimental and numerical curves for the waveguides further demonstrates that the high predictive accuracy of our approach depends primarily on accurate material characterization rather than on fabrication precision, also for two-dimensional phononic configurations. Therefore, a proper material model can significantly improve the reliability of transmission curves, especially within the band-gap frequencies, even for FDM-printed plates, which have the lowest manufacturing accuracy among polymer 3D printing techniques.

### 3.3 Three-dimensional phononics and varying temperature environment

To demonstrate the applicability and relevance of our approach to more complex phononic designs, we have applied it to study wave transmission in chiral phononic chains proposed in ref. 39. In the original work, the so-called isotactic and syndiotactic structures were manufactured from a commercial mineral fiber reinforced polyamide by selective laser sintering (SLS) and revealed substantial discrepancies between the calculated and experimental FRF curves in terms of the width and position of band gaps and the correlation between transmission peaks (see Fig. 1 in ref. 39). We have manufactured the same structures in PLA using the FDM Bambu Lab X1C 3D printer and observed that numerically estimated transmission differs negligibly from the experimental curves, with a very good match in band-gap frequencies, wave excitation and attenuation levels, and transmission peaks (Fig. S15 and Section S4.2), underlying the advances of the proposed approach.

Finally, we proved that our approach remains valid and robust under non-ambient conditions, *e.g.*, elevated temperatures, provided the constituent polymer is in the glassy regime and behaves as linear viscoelastic, as discussed in Sections S4.1 and S2.3.

## 4 Mechanical properties of additively manufactured polymers

Given the importance of elastic moduli and mass density for the accuracy of wave-dynamics predictions in phononic structures, we performed mechanical characterization of solid (100% material) and porous (57.5–95% material) parts FDM-printed from PLA and ABS.

**Fig. 6:**
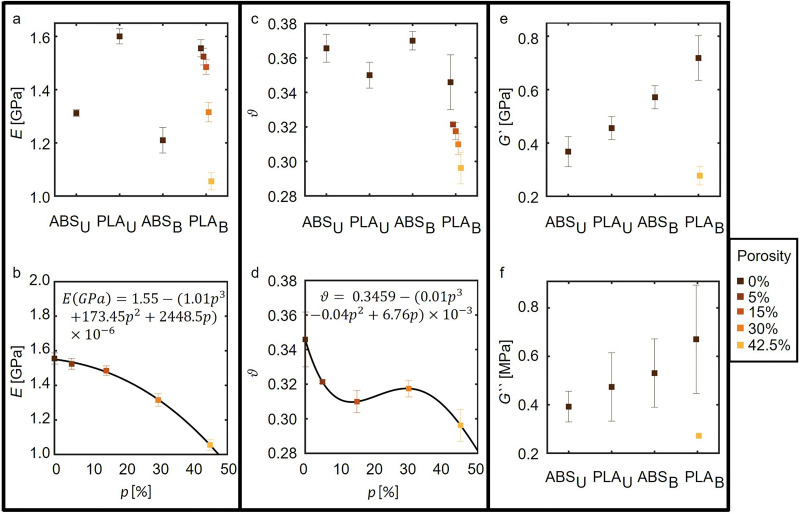
**Mechanical characteristics of FDM-printed ABS and PLA polymers using Ultimaker (U) and Bambu Lab (B).** (a) Youn's modulus and (b) Poisson's ratio of solid (0% porosity) and porous polymers. (c) and (d) Polynomial fits (*R*^2^ ≈ 1) reveal variation of (c) Young's modulus and (d) Poisson's ratio with porosity. (e) Shear storage modulus and (f) shear loss modulus measured by the dynamic mechanical analysis (DMA). The grayscale square markers indicate different porosity levels, from fully dense (black) to 42.5% porosity (white), demonstrating the combined effects of the printer platform and porosity on viscoelastic performance.

The data from tensile tests (Section 5.2.2) show minor differences in Young's moduli for dog-bone samples manufactured by two 3D printers, Ultimaker 3.0 (denoted with subscript ‘U’) and Bambu Lab X1C (denoted with subscript ‘B’) (Section 5.1). Specifically, we measured *E*^ABS^_B_ = 1.21 GPa and *E*^PLA^_B_ = 1.555 GPa for solid samples produced by the Bambu Lab and 8.4% and 3% higher values, respectively, for samples produced by the Ultimaker (Fig. 6a). Note that the measured Young moduli are almost twice lower than those from the baseline values provided by filament manufacturers for the *XY* plane (ABS_B_ ≈ 2.2 G,^77^ ABS_U_ ≈ 1.96 GPa,^78^ PLA_B_ ≈ 2.58 GPa,^79^ PLA_U_ ≈ 3.25 GPa^80^), which can be attributed to process-related effects such as interlayer porosity and imperfect adhesion. We did not observe any significant dependence on the 3D printing direction (longitudinal, lateral, diagonal) for Young's modulus and Poisson's ratio of the samples 3D-printed in the flat vertical orientation in the *XY* plane. For other orientations and print directions, this dependence exists and is discussed in Section S1.

To estimate the effect of porosity, we considered only the B samples (the Bambu Lab 3D printer) made of PLA, assuming that the other polymers would show similar trends. Expectedly, samples with increasing porosity, from 0 to 42.5%, reveal a decreasing Youn's modulus (Fig. 6b). The measured values are fitted well by a third-order polynomial function (*R*^2^ ≈ 1), allowing the estimation of intermediate values.

In contrast, Poisson's ratio *ν* appeared to be insensitive to the choice of the 3D printer for solid samples (Fig. 6c). We measured *ν*^ABS^ = 0.37 and *ν*^PLA^ = 0.346 for solid samples. Interestingly, increasing porosity to 42.5% results in a decrease of the Poisson's ratio up to 16.78% in a non-monotonic manner that can be well captured by the polynomial function with a third-order leading term (Fig. 6d). The initial decrease is followed by a slight increase in Poisson's ratio, between 15% and 35% porosity, followed by a sudden drop. This behavior can be attributed to the honeycomb infill pattern that controls porosity, as previously reported for graded honeycomb structures.^81^ However, additional analyses performed on square and auxetic (bowtie) infills reveal similar, and even more pronounced, non-monotonic variations, see Fig. S10. This suggests that the observed dependence is not unique to the honeycomb architecture, but rather a more general feature of lattice-based porosity. Therefore, porosity can be considered as a means of varying Poisson's ratio in limited ranges.

We also performed a dynamic mechanical characterization to identify the dependence of the elastic moduli of the additively manufactured polymers on frequency (Section 5.2.3). The shear storage and loss moduli reveal a slightly nonlinear behavior outside the glass transition zone (
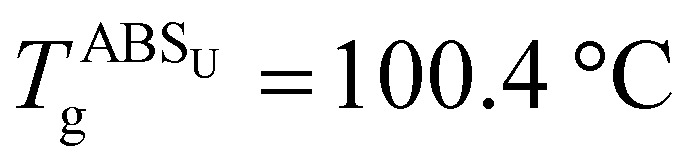
, 
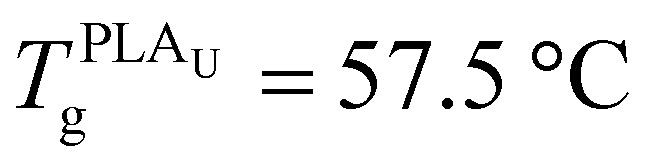
, see Fig. S3) with small viscous losses. For example, representative values of *G*′ = 714.7 MPa and *G*″ = 26.6 MPa for PLA_B_, and *G*′ = 571.5 MPa and *G*″ = 16.5 MPa for ABS_B_ were identified within the range 1 Hz to 25 kHz at reference temperature 18 °C. These values were used in numerical simulations based on a “user-defined” viscoelastic model in COMSOL Multiphysics (Section 5.4). Notably, the shear storage moduli measured by DMA consistently differ from the corresponding quasi-static values obtained from the tensile tests (Table S1). This discrepancy reflects the frequency-dependent behavior common in polymers.^82,83^ Furthermore, variations in static and dynamic moduli between the samples produced by the two 3D printers align with known differences in microstructure, crystallinity, and printing parameters that affect mechanical performance and viscoelastic behavior.^84–87^ These observations emphasize the importance of using dynamic mechanical properties for accurate modeling of wave dynamics in additively manufactured polymers.

To ensure model fidelity, the densities of the additively manufactured PLA and ABS parts were estimated using the Archimedes method (see Section 5.2.1). The measured density values were 1144 kg m^−3^ for PLA and 980 kg m^−3^ for ABS (both 3D printed with Bambu Lab) and were used in numerical simulations. These values are consistently lower than the manufacturers' specifications for the raw filament, which are 1240 kg m^−3^ and 1050 kg m^−3^, respectively. Note that using manufacturer data for material density can cause numerical results to diverge from experimental measurements, *e.g.*, by yielding higher predicted band-gap frequencies. This highlights the need to incorporate experimentally measured density values for accurate predictions of wave dynamics.

## 5 Materials and methods

### 5.1 Phononic structures manufacturing

Phononic samples were designed using COMSOL Multiphysics 6.2 and 3D printed from ABS and PLA filaments of diameter 1.75 mm with a 100% infill density and a line infill pattern. The 3D printing process used fused deposition modeling (FDM) with Bambu Lab X1 Carbon and Ultimaker 3.0 3D printers, both with a 0.4 mm-diameter nozzle. The print bed temperature was set to 100 °C for ABS and 60 °C for PLA. The nozzle speed was 40 mm s^−1^, and the print temperature was 250 °C for ABS and 200 °C for PLA. The printing direction for all samples was set to 0°. The other 3D printing parameters were set to default.

### 5.2 Material characterization

#### 5.2.1 Mass density measurements

The mass density of additively manufactured materials was measured using the Archimedes method (ASTM D792). For this, five cubic samples of 20 mm size were manufactured following the procedure in Section 5.1 and kept in ambient laboratory conditions (18 °C, 40% relative humidity) for at least 24 hours before measurements. A high-precision weighing balance (±0.1 mg resolution) was used first to measure the dry mass of the cubes, *m*_a_, and then their apparent mass, *m*_w_, when the cubes were fully immersed in deionised water. Special care was taken to avoid air bubbles adhering to the sample in water. The mass density of each sample was calculated using the formula:1
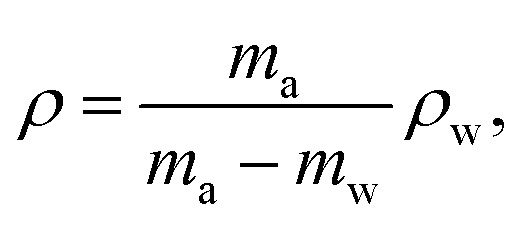
where *ρ*_w_ is the density of deionized water assumed to be 1000 kg m^−3^ at 18 °C. The final values are the average of three independent measurements.

#### 5.2.2 Quasi-static tensile testing

Uniaxial tension tests were conducted on additively manufactured dog-bone samples using a universal testing machine (UTM, Instron 34SC-1) with a maximum load capacity of 1 kN (ISO 527-2 standard). Three samples per material were tested. The test parameters, including a 60 ramp time, a 10 recovery time, and a 5 mm min^−1^ loading speed, were used to record 10 data points per second for a maximum force of 950. The tests were carried out at ambient conditions (18 °C, 40% of relative humidity).

A random speckle pattern (with a global residual value <0.1) was applied to one face of the samples to measure the strain field using a Q400 DIC system (Limess, Germany) equipped with two high-resolution cameras. The UTM and DIC systems were calibrated according to the manufacturer's guidelines for accurate strain measurements. Commercial software Istra4D 4.6 (Dantec Dynamics GmbH) was used to analyze the captured images and estimate Poisson's ratio from the measured strain data.

#### 5.2.3 Dynamic mechanical analysis

Dynamic mechanical analysis (DMA) was performed using a DMA 8000 analyzer (PerkinElmer Inc., USA) to measure the storage and loss elastic moduli of additively manufactured parts. First, the glass transition temperature *T*_g_ was estimated using differential scanning calorimetry (DSC) and set as an upper temperature limit.

Three rectangular ABS and PLA strips of dimensions 16.8 mm × 7.8 mm × 0.6 mm were manufactured with a linear 100% in-fill pattern aligned at 0°. After mounting the sample in a single cantilever configuration, a frequency sweep ranging from 0.1 Hz to 100 Hz was employed under temperatures varying between −20 °C and 70 °C. The heating rate was 2 °C min^−1^, and the temperature increased in 5 °C increments with a 5 minute isothermal pause at each step. To extract frequency-dependent elasticity moduli beyond the tested range, time-temperature superposition (TTS) was applied. For this, the curves of the shear storage (*G*′) and loss (*G*″) moduli measured at different temperatures were shifted using the Williams–Landel–Ferry (WLF) equation, aligning all data to a reference temperature of 18 °C.^40,82,83,88,89^

### 5.3 Ultrasonic transmission tests

Wave transmission in phononic samples was measured in pitch-catch tests. For this, an arbitrary waveform generator (AWG, NETBOX DN2.653-16, Spectrum) and the commercial software SBench6 were used to linearly sweep a signal over the frequency range estimated in numerical simulations (Section 5.4) and to record the dynamic response at acquisition points. The generated signal was amplified (20× voltage gain) using a power amplifier (Bruel & Kjaer Type 2718) and sent to a shaker (Bruel & Kjaer Type 4810). The test sample was connected to the shaker using a 10–32 UNF screw. This screw was held in a custom-designed attachment fabricated from the same material (as the test sample) to minimise impedance mismatch. The attachment was then bonded to the test sample using a cyanoacrylate adhesive (Precision Super Glue-3, Loctite). A laser Doppler vibrometer (LDV with a compact sensor head, OFV-534, and an LDV controller, OFV-5000, from PolyTec, Germany) was used to acquire the transmitted signal at the desired locations. Velocity measurements were obtained using a VD-09 digital velocity decoder. For each sample, three types of transmission tests were performed: one measurement without a generated signal to record ambient noise, a second measurement at the excitation point above the shaker, and a third measurement at the acquisition point. Experimental transmission curves were obtained as a difference between the signals measured at acquisition and excitation points. The results are averaged per 10 measurements. Tests were conducted on two samples of each configuration to verify consistency. The effects of various excitation conditions are discussed in Section S2.2 and shown in Fig. S5.

### 5.4 Numerical modelling

The dispersion and transmission properties of the analyzed phononic structures were studied using commercial finite element software COMSOL Multiphysics v6.2.^40,90^

#### 5.4.1 Viscoelastic model

The material behavior is modeled as linear viscoelastic, assuming small deformations and strain-independent material properties, see Section S2.3. To properly capture the frequency-dependent behavior of the elastic moduli of constituent materials, a user-defined viscoelastic model was implemented in the linear elastic domain of the “Solid Mechanics” module for both dispersion and transmission studies. The model used elastic parameters (*E* and *ν*) obtained from quasi-static tests, and viscoelastic properties (*G*′ and *G*″) extracted from DMA. Frequency-averaged values of *G*′ and *G*″ (from 1 Hz to 25 kHz) were separately specified within the model.

#### 5.4.2 Dispersion analysis

The structural periodicity was replicated by applying Floquet–Bloch periodic conditions at the lateral faces of a single unit cell. The unit cell domain was automatically meshed with free tetrahedral elements at a “finer” resolution, calibrated for general physics. The resulting mesh density was adequate across all models, as the element size was well below the smallest wavelengths associated with the unit cell sizes studied. Specifically, for both shear and longitudinal elastic waves, the minimum wavelength corresponding to each unit cell geometry was discretised into more than 18 mesh elements per wavelength. For example, in the case of PLA (*E* = 1555 MPa, *ν* = 0.346, *ρ* = 1144 kg m^−3^), the shear wave velocity is approximately 711, yielding a minimum wavelength of ∼35.5 mm at the maximum analysed frequency of 20 kHz. The adopted mesh (maximum element size ≈3.3 mm) corresponds to ∼11 elements per wavelength. This number exceeds the standard meshing criterion of six to ten elements per wavelength, ensuring accurate representation of wave propagation phenomena.

The *ω*(*k*) approach was used to calculate the real and imaginary branches of dispersion curves.^36,58,91,92^ For real components, the wavenumber *k*_*x*_ was discretised into 300 equally spaced values and swept along the boundary *ΓX* of the Brillouin zone spanning from 0 to π/*a*, while keeping *k*_*y*_ = 0 and *k*_*z*_ = 0. The ARPACK solver was utilized to solve an eigenvalue problem resulting from the dispersion relation.

The first 15 eigenfrequencies were calculated, and the corresponding eigenvectors were averaged over the unit cell volume to estimate the polarization factor *P*:2
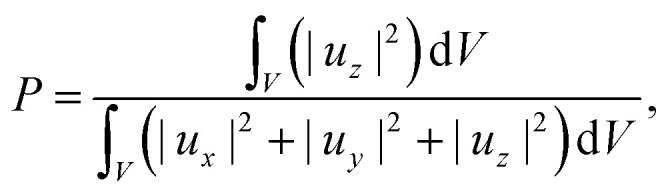
to distinguish between the in-plane (*P* = 0) and out-of-plane (*P* = 1) mode polarization. Here *V* is the volume of the unit cell, *u*_*x*_, *u*_*y*_, and *u*_*z*_ are the displacement components along *x̂*, *ŷ*, and *ẑ* axes, respectively.

To compute the imaginary part of the wavenumber, we introduced *k* = −*ik*_*x*_ as a modified *x*-component of the Floquet–Bloch vector. The other simulation settings were identical to those described above, except the values of *k*_*x*_ were swept between 0 and 3π/*a* due to the lack of periodicity of the solutions in the imaginary plane.^58^

#### 5.4.3 Transmission analysis

We designed rectangular (7 × 1 unit cells) and square (7 × 7 unit cells) finite-size structures with stress-free boundary conditions. We did not employ any perfectly matched layer (PML) in the simulations. Preliminary tests revealed no significant impact on the results with the inclusion of PML. More critically, the simulations were designed to replicate the experimental conditions as closely as possible. Hence, all key geometric and boundary details, including the mechanical screw used to mount the test sample on the shaker, were modeled. We also considered the gravitational force proportional to the structural weight. This approach provided a realistic representation of boundary interactions, eliminating the need for PML in this study. Each polymer structure was assigned viscoelastic material properties (Section 5.4.1) and excited by an out-of-plane displacement of 1μ at the center of the second or left-bottom (Fig. S6) unit cell, respectively.

The transmission losses (*T*_*l*_) were estimated as a ratio of the amplitudes of out-of-plane transmitted (*u*_*z*_) and incident (*A*) waves, for a frequency range of 0.1 kHz to 25 kHz, discretized into 2000 evenly spaced intervals.3
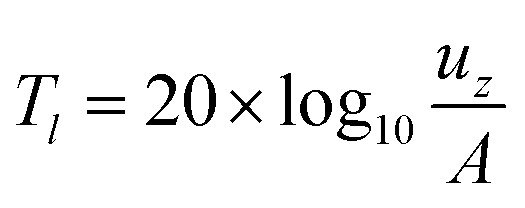
However, due to variations in unit cell sizes across configurations, transmission results are presented up to a normalized frequency of f* = 0.8 to allow for consistent comparison. The drops exceeding 20 dB in the transmission *vs.* frequency graphs are classified as frequency band gaps.

## 6 Conclusions

Accurate prediction of wave propagation in additively manufactured phononic structures is crucial, given their tremendous potential for vibration suppression, waveguiding, frequency filtering, energy harvesting, and other applications that require precise knowledge of their dynamic response.

In this work, we presented a framework for reliably estimating wave characteristics in polymer phononic structures of any design using standard equipment, while maintaining excellent agreement with experimental data. We show that the high predictive capability of our approach is independent of the structural dimensionality (1D, 2D, or 3D), the integrated wave manipulation mechanism (Bragg scattering, local resonance, or inertia amplification), the constituent material (PLA, ABS, or resin), and additive manufacturing technology (FDM or SLA). It is also robust to varying environmental conditions, provided constituent materials remain in the glassy state.

By using FDM-manufactured samples, characterized by low printing resolution and defect-rich final parts, we demonstrated that discrepancies between experimental and theoretical results for wave characteristics in phononic structures primarily arise from improper material characterization, particularly viscous losses, rather than from fabrication-induced geometric imperfections as commonly attributed. Although the reported viscous losses are small, we showed that ignoring them leads to incorrect predictions of band-gap frequencies, attenuation levels, and transmission peaks, even at comparatively low frequencies. Our results establish accurate material characterization as a central requirement for predictive numerical modeling of wave propagation in additively manufactured phononic materials. However, at higher frequencies, when the wavelength is comparable to characteristic sizes of manufacture-driven defects, the role of anisotropic material response and imperfect bonding cannot be ignored anymore and may be substantial. We also note that the presented framework is applicable to linear viscoelastic polymers, while nonlinear or large-amplitude excitations require a separate treatment.

Furthermore, we proposed to use small variations in geometric and structural features, such as the inclination angles of thin ligaments or variable internal porosity, to fine-tune the dispersion and transmission characteristics of additively manufactured polymer phononics. Controlled changes in unit-cell geometry lead to systematic shifts in band-gap position, bandwidth, and transmission characteristics. These variations are consistently captured by our experiment-based viscoelastic material characterization and can be used to implement exotic functionalities, such as direction-dependent propagation or waveguiding.

Our results advance the field of phononics in several directions. First, material characterization is shown to be an essential step for accurate predictions of wave dynamics in any phononic structure. Second, low-resolution FDM phononic structures offer a robust and reliable platform for testing exotic dynamic functionalities, provided that proper material properties, including viscous losses, are used. Third, our approach can be easily extended to other additive manufacturing techniques based on vat photopolymerization, powder bed fusion, material or binder jetting, *etc.*, to accelerate the adoption of phononic concepts across numerous fields. Therefore, our work indicates that polymer phononic materials can serve as predictable systems for wave engineering and opens the door for their use in applications implying vibration isolation, signal manipulation, ultrasonic communication, energy harvesting, sensing, structural health monitoring, and others.

## Author contributions

Sidharth Beniwal: conceptualization (equal), methodology (equal), software (lead), data curation (lead), investigation (lead), validation (lead), formal analysis (equal), visualization (lead), writing – original draft (equal), writing – review and editing (equal). Ranjita K. Bose: methodology (supporting), formal analysis (supporting), supervision (supporting), resources (supporting), writing – review and editing (supporting). Anastasiia O. Krushynska: conceptualization (equal), methodology (equal), funding acquisition (lead), visualization (supporting), project administration (lead), resources (lead), supervision (lead), writing – original draft (equal), writing – review and editing (lead).

## Conflicts of interest

The authors declare no conflict of interest.

## Supplementary Material

MH-013-D6MH00395H-s001

## Data Availability

Data for this article, including COMSOL models, raw experimental data (tensile testing, Poisson's ratio, TGA, DSC, DMA), simulation outputs, and processed datasets used for figure generation, are available at DataverseNL: https://doi.org/10.34894/ACD7BE. The supplementary information contains details of the material characterization workflow, finite-element modelling procedures, and transmission experiments. It also includes additional results demonstrating the predictive capability of the proposed framework across different phononic architectures, wave-control mechanisms, constituent materials, manufacturing techniques, and environmental conditions, together with supporting figures, tables, and quantitative comparisons between numerical and experimental data. See DOI: https://doi.org/10.1039/d6mh00395h.
