# Supplementary material for: Predictive wave engineering in polymer phononic materials via viscoelastic–geometric coupling
Source: Mater Horiz. 2026 Jun 3;13(15):7494–509. doi: 10.1039/d6mh00395h (PMC13262411; doi:10.1039/d6mh00395h)
Supplement: MH-013-D6MH00395H-s001 [file MH-013-D6MH00395H-s001.pdf]

Supplementary Information (SI) for Materials Horizons.
This journal is © The Royal Society of Chemistry 2026

Journal Name

ARTICLE TYPE

Cite this: DOI: 00.0000/xxxxxxxxxx

Supplementary Material: Predictive Wave Engineering in Polymer Phononic Materials via Viscoelastic–Geometric Coupling†

Sidharth Beniwal,*[a] Ranjita K. Bose,[a] and Anastasiia O. Krushynska[a]

*

S1 Mechanical Characterization of FDM PLA and ABS 3D-printed by Bambu Lab and Ultimaker 3D printers

The mechanical, thermal, and viscoelastic characterization of 3D-printed PLA and ABS fabricated using FDM Bambu Lab X1C and Ultimaker S3 3D printers was performed using quasi-static tensile tests, temperature-dependent TGA and DTG, and dynamic DMA measurements.

Quasi-static Young's modulus E was measured on dog-bone samples under tensile load, Poisson's ratio ν was calculated from the strain measurements using DIC, and shear modulus G was estimated as $G = E/[2(1+\nu)]$. The quasi-static mechanical response is shown in Fig.S1, including stress–strain curves and transverse–axial strain relationships for solid and porous specimens.

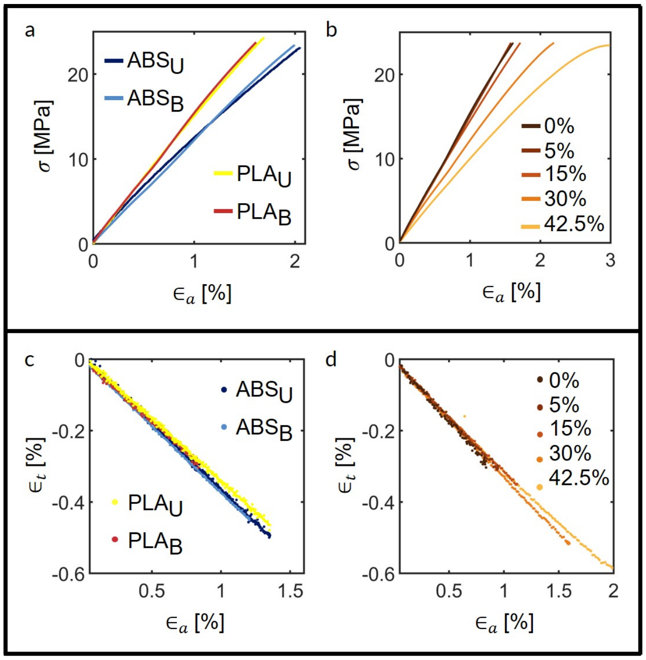

Fig. S1 Quasi-static mechanical characterization. (a) Stress–strain curves for solid ABS and PLA dog-bone samples made by the Ultimaker and Bambu Lab 3D printers. (b) Stress–strain curves for PLA$_B$ dog-bone samples with varying porosity levels. (c) Transverse versus axial strain curves for solid ABS and PLA dog-bone samples 3D printed on the Ultimaker and Bambu Lab. (d) Transverse versus axial strain curves for PLA$_B$ dog-bone samples with different porosity levels.

The effects of print orientation and raster angle on the mechanical properties of PLA and ABS (Fig. S2) were estimated by testing dog-bone samples 3D-printed in three build orientations (flat, on-edge, and upright) and with raster angles of 0°, 45°, and 90°. The print parameters were identical to those described in Section 5.1. To enable direct comparison across materials and orientations, the measured Young's moduli are presented in normalized form using manufacturer-reported in-plane (E_{xy}) and out-of-plane (E_z) reference values. These values are $E_{xy_{\mathrm{ref}}} = 2580\,\mathrm{MPa}$, $E_{z_{\mathrm{ref}}} = 2060\,\mathrm{MPa}$ for PLA[1] and $E_{xy_{\mathrm{ref}}} = 2200\,\mathrm{MPa}$, $E_{z_{\mathrm{ref}}} = 1960\,\mathrm{MPa}$ for ABS.[2] The stress–

[a] Faculty of Science and Engineering, Engineering and Technology Institute Groningen (ENTEG), University of Groningen, Groningen, The Netherlands. E-mail: s.beniwal@rug.nl
* *Correspondance E-mail: s.beniwal@rug.nl

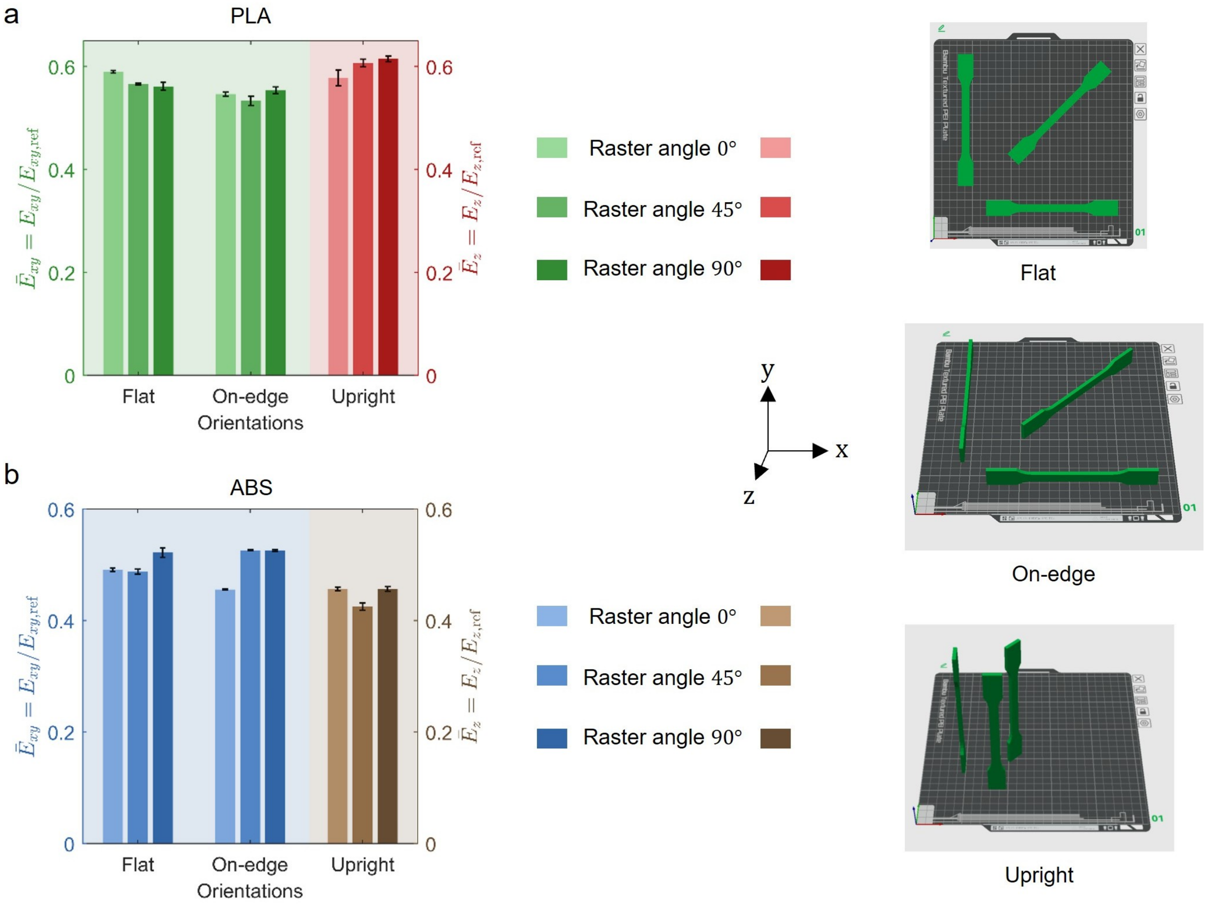

Fig. S2 Orientation-dependent normalized tensile Young's moduli of FDM-printed polymers. The moduli values for (a) PLA and (b) ABS dog-bone specimens (ISO 527) fabricated in three build orientations (flat, on-edge, and upright) and with raster angles of 0°, 45°, and 90°. The references for normalization are manufacturer-reported in-plane (E_{xy}) and out-of-plane (E_z) Young's moduli. The sliced images of the build orientations are shown on the right.

strain and transverse strain curves shown in Figs. S4a-b for the flat-printed samples with raster angle 0° reveal directional stiffness and dependence of Young's modulus and Poisson's ratio on build orientation, filament alignment, and interlayer bonding.

The thermal and viscoelastic responses of the materials are summarized in Fig.S3, which presents thermogravimetric analysis (TGA), differential scanning calorimetry (DSC), and DMA measurements for PLA and ABS samples, showing the frequency-dependent shear storage and loss moduli. The values of the quasi-static elastic and viscoelastic elasticity moduli is given in Table S1.

Table S1 Elastic and viscoelastic properties of PLA and ABS samples 3D printed in the XY plane with raster angle 0° using the Bambu Lab X1C and Ultimaker S3 FDM 3D printers.

Material	Elastic			Viscoelastic	
	E [GPa]	ν [-]	G [GPa]	G' [GPa]	G'' [MPa]
ABS_U	1.31	0.37	0.48	0.37	9.62
ABS_B	1.21	0.37	0.44	0.57	16.54
PLA_U	1.60	0.35	0.59	0.46	13.68
PLA_B	1.56	0.346	0.58	0.72	23.53

S2 Wave transmission in FDM 3D-printed phononic structures

S2.1 Anisotropic vs. isotropic material model

To assess the impact of the anisotropy of the mechanical properties of 3D-printed FDM polymers, we numerically estimated wave transmission for the straight thin-ligament geometry (Fig. 2b) using both isotropic and orthotropic material models. The curve predicted based on the orthotropic model with the experimentally measured mechanical properties better matches the experimental data

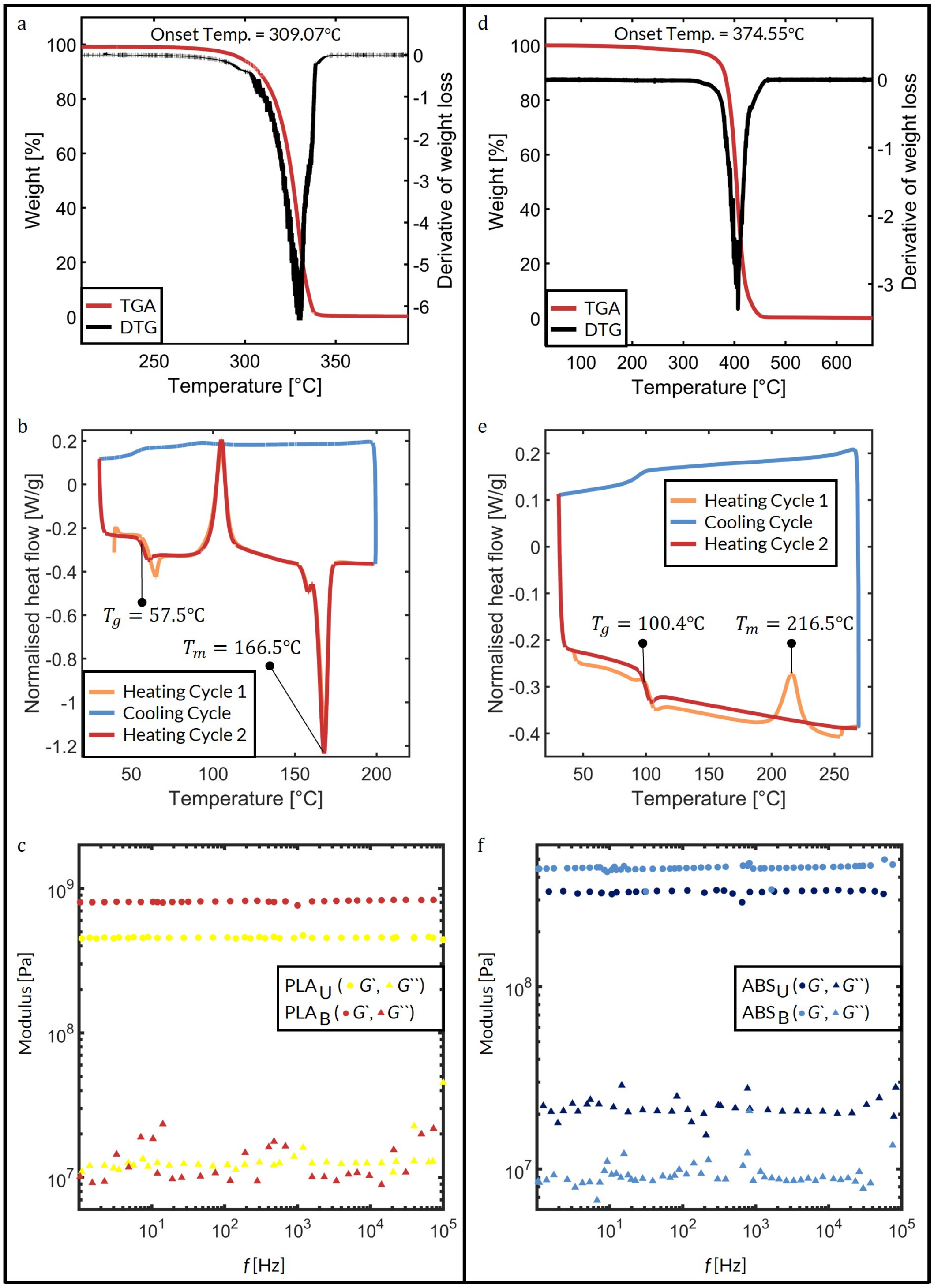

Fig. S3 Thermal and viscoelastic characteristics of 3D-printed (a-c) PLA and (d-f) ABS: (a, d) TGA and DTG curves, (b, e) DSC heating–cooling cycles, and (c, f) DMA frequency-dependent shear storage/loss moduli for the Ultimaker and Bambu Lab samples.

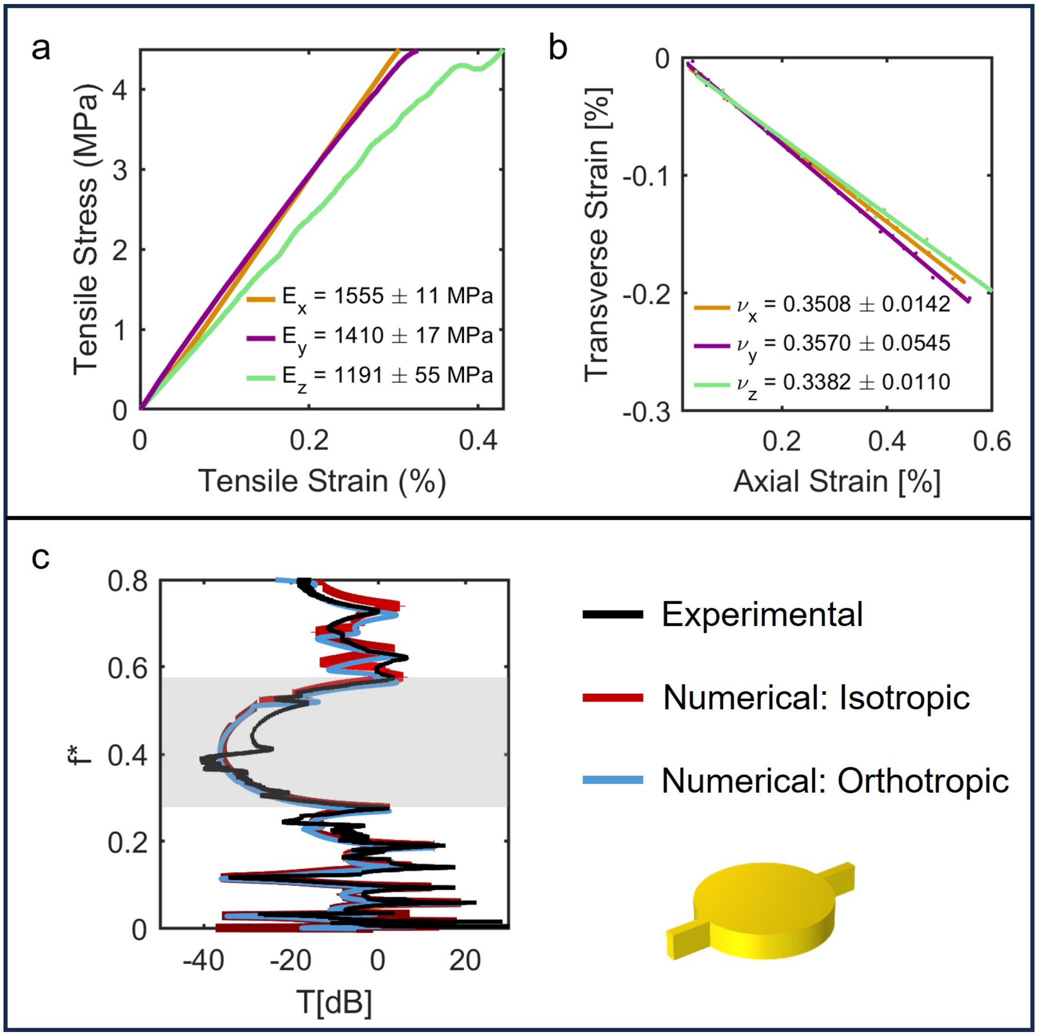

Fig. S4 Influence of anisotropy on mechanical response and wave transmission. (a) Tensile stress–strain curves for specimens printed in the three orientations described above, with a raster angle of 0°. (b) Transverse strain versus axial strain for the same orientations; mean values and corresponding standard deviations are indicated in the legends. (c) Comparison of experimental transmission results for the straight thin-ligament geometry with numerical predictions obtained using the original isotropic model and an orthotropic model calibrated from the mechanical data in panels (a) and (b). All results are presented for PLA printed on Bambu Lab X1C.

compared to that based on the isotropic material at frequencies above the band gap (Fig. S4c and Table S2). At lower frequencies, the differences between the three curves are negligible. Therefore, accounting for anisotropic mechanical behavior in FDM-printed phononic structures can be essential for fine-tuning wave-propagation characteristics at high frequencies.

S2.2 Effects of wave excitation sources

To assess the influence of excitation conditions on the transmission response, we have tested three actuation systems (Fig. S5). Specifically, we used two electrodynamic shakers with distinct dynamic capabilities and a d_{33}-based Macro Fiber Composite (MFC) actuator. A high-performance electrodynamic shaker (Brüel & Kjær Type 4810) and a lower-power laboratory vibration generator (Philip Harris B8H30701) enable a direct comparison between the excitation conditions with different dynamic characteristics. The high-performance shaker delivers a higher excitation force (up to 10 N) and a relatively constant peak-to-peak displacement of approximately 4 mm across the frequency range of interest, enabling stable, broadband energy input. In contrast, the Philip Harris generator operates at much lower forces (up to 1.5 N) and exhibits a frequency-dependent displacement response, with peak-to-peak amplitudes up to 8 mm at 1 Hz. This results in different excitation conditions, including variations in input force amplitude and spectral uniformity. Despite these differences, the experimentally measured band gaps remain consistent across all configurations, indicating that wave attenuation is predominantly governed by structural dynamics rather than by the specifics of the excitation source. Minor variations in amplitude and spectral features can be attributed to system-level effects, including fixture compliance and reflections for the boundaries.

Table S2 Quantitative comparison between the experimental results and numerical predictions (based on the isotropic and orthotropic models) for the straight thin-ligament geometry (Fig. 2b), including band-gap mid-frequency, band-gap width, and attenuation depth, along with their respective deviations from experimental values.

Case	Band-gap position			Band-gap characteristics			
	Mid-freq [kHz]	Deviation [Hz]	Deviation [%]	BG width [kHz]	BG width [%]	Attenuation depth [dB]	Deviation [%]
Experimental	9.449	0	0	6.61	100	−44.57	0
Numerical (Isotropic)	9.434	−20	−0.2	6.67	100.9	−47.05	−5.6
Numerical (Orthotropic)	9.269	−180	−1.9	6.57	99.4	−45.54	−2.2

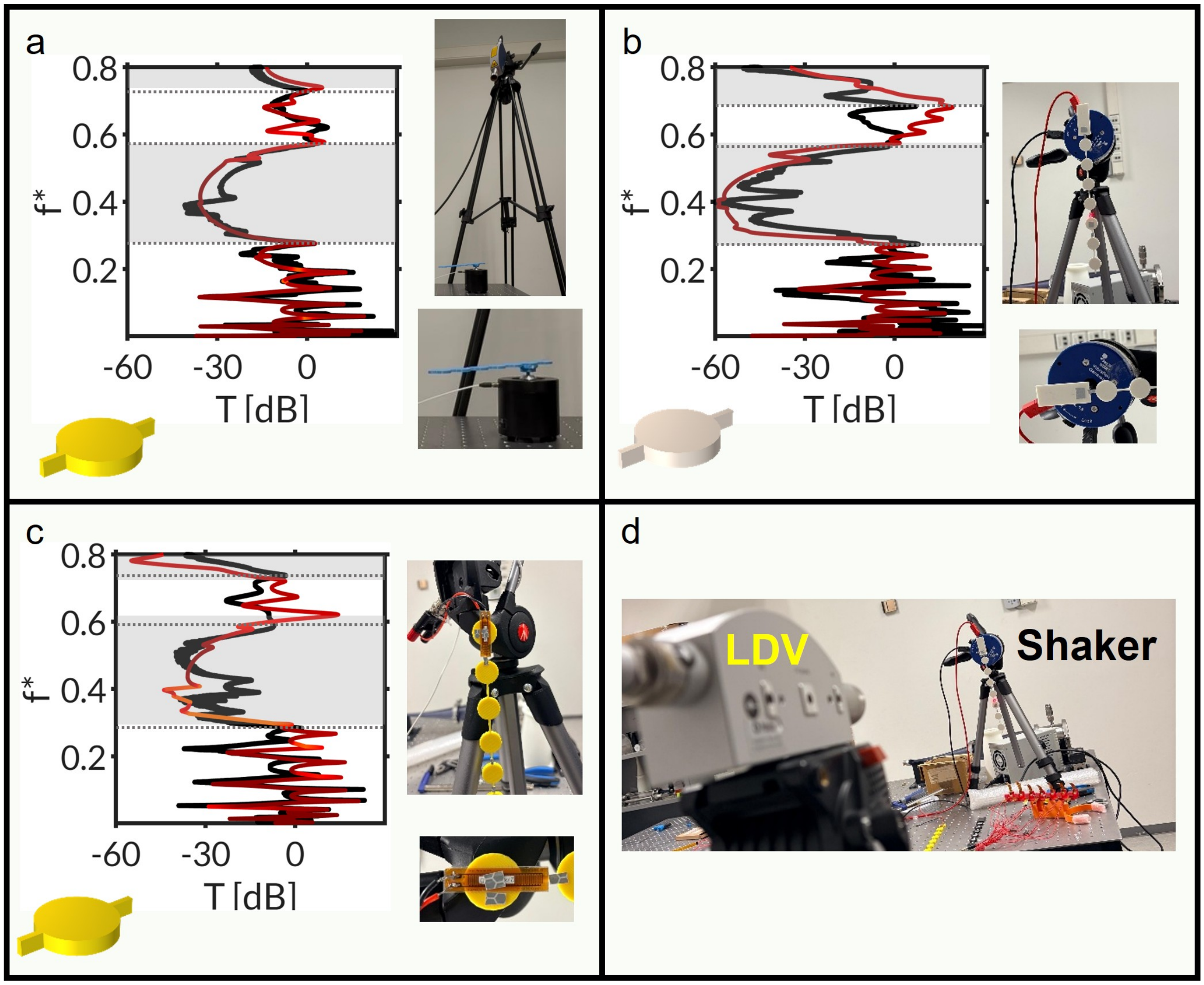

Fig. S5 Transmission measurements under different excitation and measurement configurations. Transmission results for thin-ligament geometries (Fig. 1a) excited using (a) Brüel & Kjær Type 4810 shaker, (b) Philip Harris vibration generator (B8H30701), and (c) d_33 based P1-type macro fiber composite (MFC) actuator (Smart Materials). (d) Schematic of the experimental setup used for transmission measurements with a laser Doppler vibrometer (LDV). Grey shaded regions indicate numerically predicted band gaps, while grey dashed lines denote the bounds of experimentally observed band gaps. Insets in (a-c) show zoomed views of the respective excitation configurations.

The excitation configuration is illustrated in Fig. S6. The numerical model replicates the experimental excitation conditions, with the structure subjected to out-of-plane harmonic loading consistent with the applied boundary conditions in the measurements. The dynamic response is measured experimentally using a non-contact, high-resolution laser Doppler vibrometer (LDV). This setup ensures a consistent basis for comparison between numerical simulations and experiments, enabling direct validation of the modeled transmission behavior.

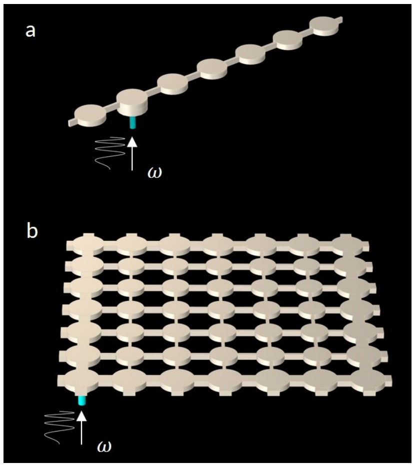

Fig. S6 Excitation configuration for polymer phononic materials. Schematic representations of (a) polymer chain and (b) phononic plate excited by an out-of-plane harmonic displacement of amplitude $1\,\mu\mathrm{m}$.

S2.3 Linear vs. Nonlinear viscoelasticity

Here, we assume that FDM 3D-printed polymers are thermorheologically simple that enables the application of the time-temperature superposition principle to DMA data, implying that a change in temperature is equivalent to a shift in viscoelastic moduli along the log-frequency or time axis. Such polymers exhibit linear viscoelastic response under small strain amplitudes.

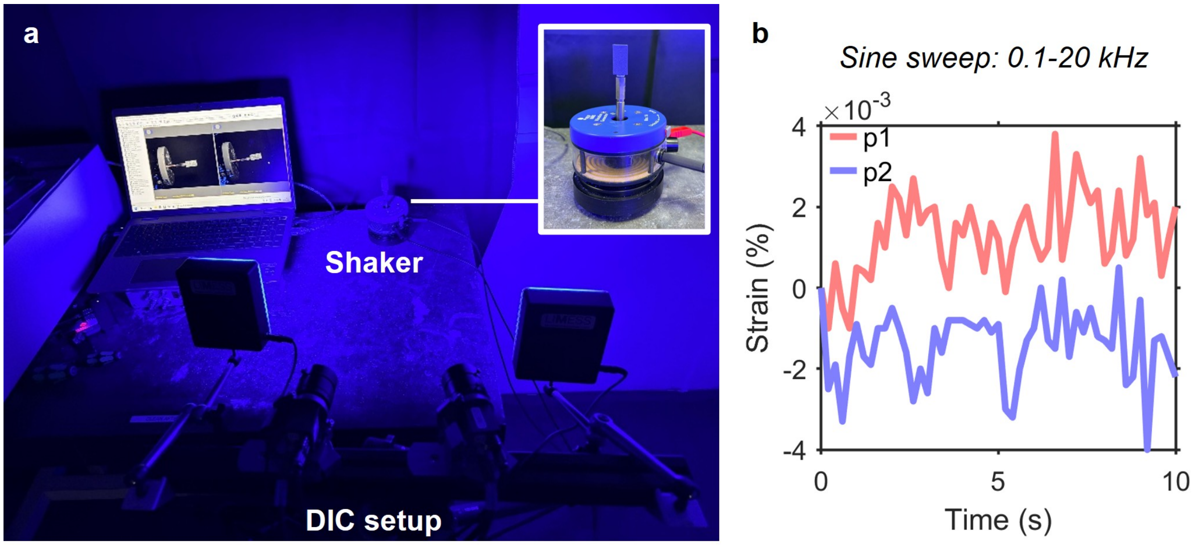

Fig. S7 (a) Full-field strain distribution measured using digital image correlation (DIC) on a cuboid-shaped PLA specimen subjected to harmonic excitation in the frequency range 0.1–20 kHz using a Phillip Harris vibration generator at a maximum current of 1 A. The strain amplitudes were extracted from the same excitation signal used to analyze the transmission behavior of the finite-sized structure in the original manuscript. A magnified view of the PLA specimen mounted on the shaker is also presented in (a). (b) Temporal evolution of the principal strains (%) under harmonic loading. The specimen was excited with a 10-second frequency sweep from 0.1 to 20 kHz, while strain data were obtained from 50 DIC images captured at 5 images per second.

To confirm the validity of the assumed linear viscoelastic material response in experimental tests, we applied maximum possible excitation on a test sample and measured full-field strain using digital image correlation (DIC) (Fig. S7). A cuboid PLA specimen was subjected to harmonic excitation in the frequency range from $0.1\,\mathrm{kHz}$ to $20\,\mathrm{kHz}$ using a Philip Harris vibration generator, representing a conservative scenario with relatively strong excitation. The excitation was applied as a $10\,\mathrm{s}$ frequency sweep, while strain fields were recorded at 5 frames per second. The measured principal strain amplitudes remained on the order of $10^{-3}\%$, with maximum values of $+0.0038\%$ and -0.004%. These strain levels are more than two orders of magnitude lower than the $0.1\,\%$ strain amplitude used in the DMA tests, where the material response was confirmed to be linear viscoelastic. The temporal evolution of the strain field further demonstrates a consistent, stable response with no indication of nonlinear effects. These results therefore provide experimental evidence that the strain amplitudes in the wave propagation experiments remain within the linear viscoelastic regime, thereby justifying

the use of a linear viscoelastic model in the numerical simulations.

S2.4 Effects of the number of unit cells

The number of unit cells in the finite structures in this study was limited to 7 due to the print bed size on the Bambu X1C printer. This number is, however, sufficient to capture the dynamic response of periodic structures. To illustrate this and exclude the effects of eventual size effects, we have calculated transmission responses for a smaller (5 unit cells) and three larger samples (9, 11, and 13 unit cells). Figure S8 shows that the curve for the 7-unit-cell specimen is indistinguishable from those of the larger samples by differing only in the level of attenuation within the band gap. Additionally, the transmission drop, corresponding to the band gap, occurs exactly at the frequencies predicted by the dispersion analysis for a truly periodic system (shaded region).

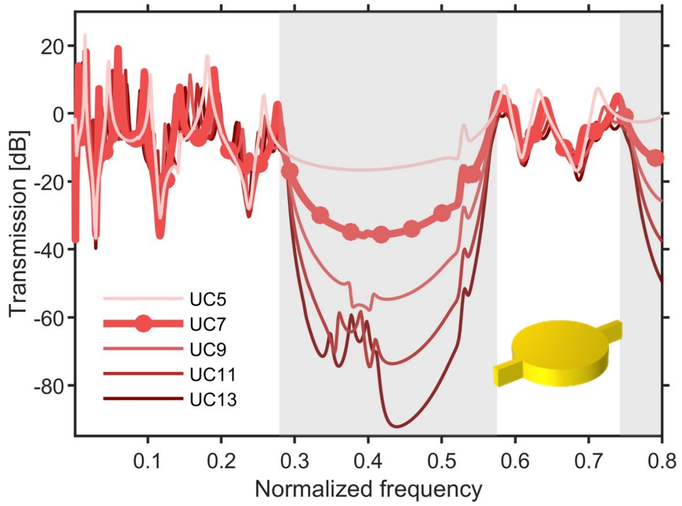

Fig. S8 Numerical transmission spectra as a function of normalized frequency for different numbers of unit cells in the thin ligament geometry shown in Fig.2b of the manuscript.

S2.5 Effects of structural internal porosity

The proposed approach to introducing internal porosity imposes an upper limit of 53.4%. Figure S9 illustrates the thin-ligament geometry at this limit, realized as a shell structure (without infill) with a wall thickness of 0.8 mm. While higher porosity can be achieved by reducing wall thickness using higher-resolution techniques (e.g., SLA or SLS), this is constrained by quickly decreasing structural rigidity and practical challenges in removing trapped resin or powder from enclosed cavities.

The choice of infill pattern also influences the variation in the structure's effective Poisson's ratio with increasing porosity, as dis-

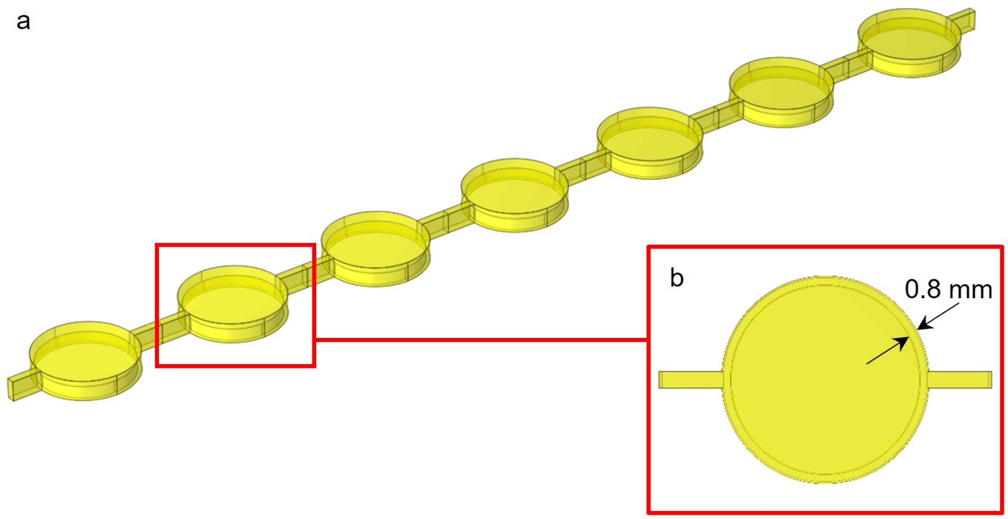

Fig. S9 (a) Thin-ligament geometry with maximum internal porosity, realized as a shell structure without internal infill and a wall thickness of 0.8 mm. (b) Top view of the corresponding unit cell.

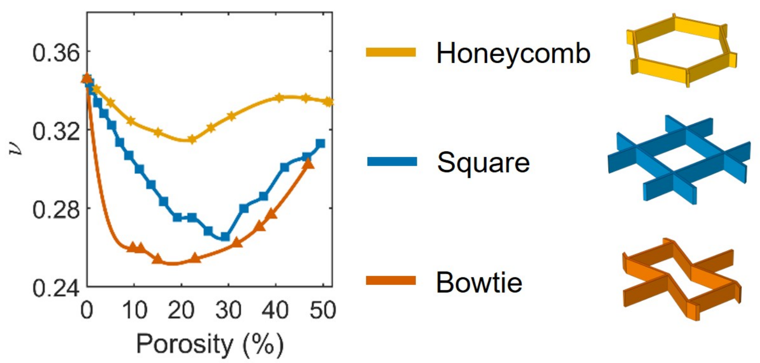

Fig. S10 Variation of Poisson's ratio with increasing internal porosity is non-monotonic for three different infill patterns: honeycomb, square, and bowtie (auxetic), with the corresponding unit cells shown on the right. The out-of-plane thickness (3.2 mm) and side length (4 mm) are kept constant for all geometries, while the in-plane wall thickness is varied to control the internal porosity.

cussed in the main text. To confirm that these variations are not specific to a particular infill pattern, we have analyzed the designs with honeycomb, square, and auxetic (bowtie) infills and observed that in all the cases, the effective Poisson's ratio depends on internal porosity non-monotonically, as shown in Fig. S10. This confirms the statement that internal porosity can be used to fine-tune Poisson's ratio of phononic structures.

S3 Band structure analysis of phononic materials

S3.1 Effects of ligament rotation angles and internal porosity

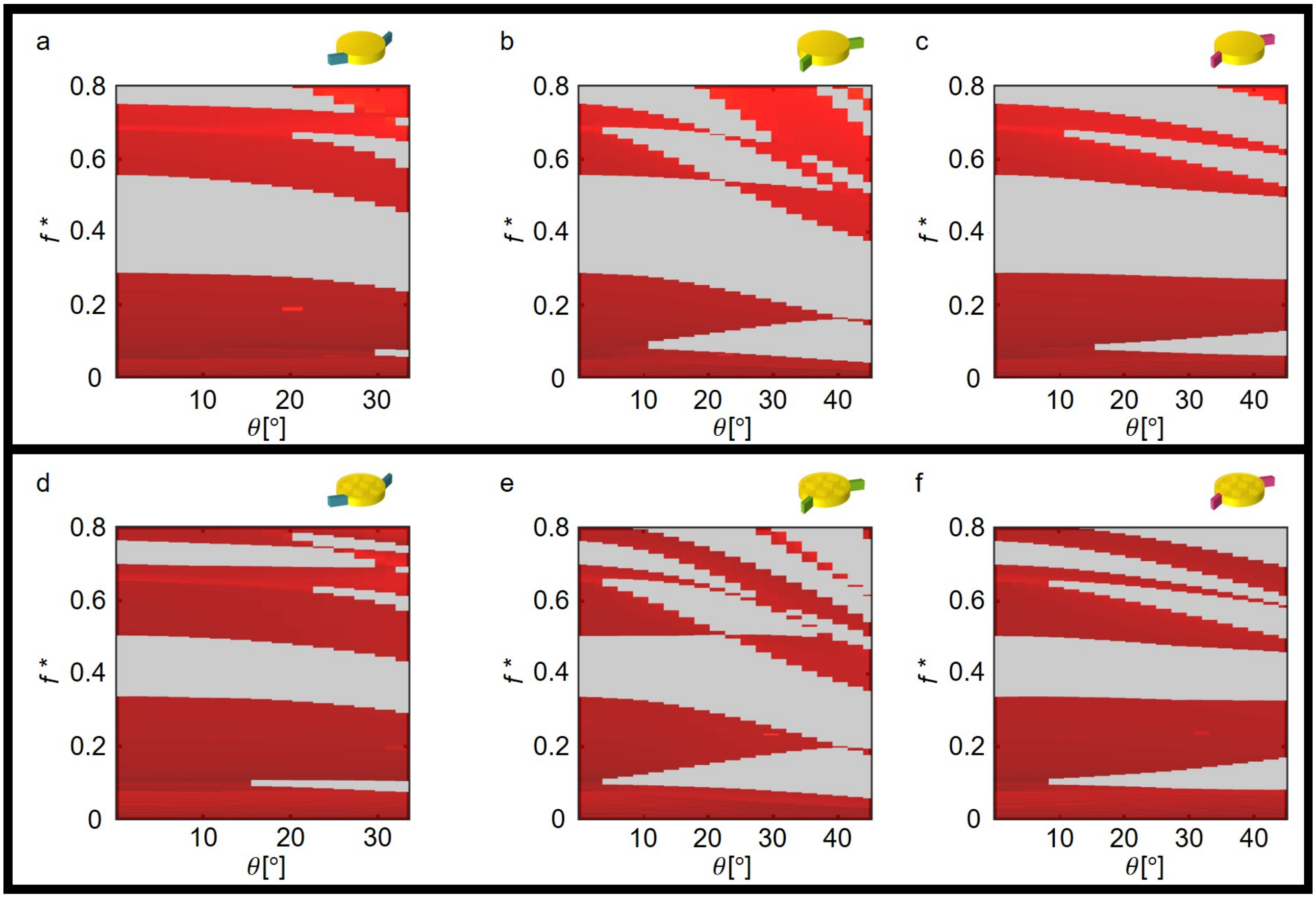

Fig. S11 Condensed band structure diagrams for the unit cells with varying ligament rotation angles shown in the insets. Results are presented for (a,d) R_1, (b,e) R_2, and (c,f) R_3 configurations (see Fig. 1), for (a–c) solid and (d–f) porous (42.5% porosity) designs.

Figure S11 shows condensed band structure diagrams for the disk-ligament unit cells with three different approaches to the ligament rotation for solid (100% material) and porous (47.5% material) designs. As discussed in Section 2.1, varying the ligament rotation across the configurations in Fig. 1b introduces kinematic constraints that modify the effective stiffness and inertia of the unit cell, resulting in a systematic shift of band gaps toward lower frequencies. The internal porosity further alters the dispersion characteristics by reducing the effective stiffness and modifying the band-gap width and position. The comparison between the solid and porous designs demonstrates that the combined use of rotation and internal porosity provides an additional degree of control over wave propagation behavior.

S3.2 Viscosity-induced frequency shifts

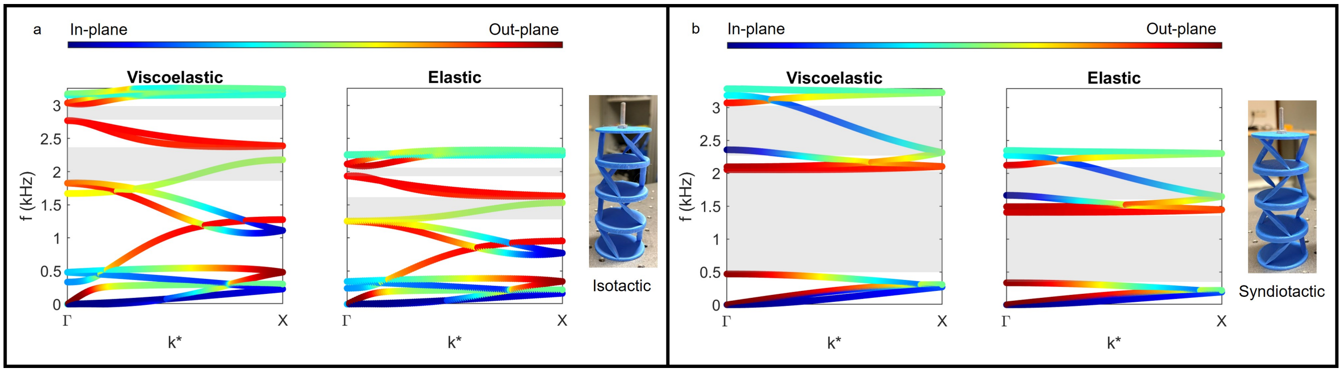

Fig. S12 Band structure diagrams based on viscoelastic and purely elastic material models for (a) isotactic and (b) syndiotactic geometries from Ref.[3], shown in the insets on the right-hand side. The band gaps are highlighted in grey.

Viscoelastic materials exhibit a frequency-dependent dynamic response, influencing the band structure diagrams of phononic materials. In particular, neglecting viscous losses alters the material stiffness, shifting the frequencies of propagation and attenuation bands.

The main text reports that neglecting viscosity in disk-ligament phononic designs results in an approximately 31% frequency downshift in the band-gap frequencies. To reveal the presence of similar shifts in other phononic geometries, we estimated band-gap frequencies in two three-dimensional chiral architectures – isotactic and syndiotactic phononic structures reported in Ref.[3].

The calculated results shown in Fig. S12 indicate substantial shifts of all bands to lower frequencies for an elastic material model compared to the data for the viscoelastic (Bambu PLA) material. Despite the distinct structural geometries, neglecting viscosity results in comparable shifts in the mid-frequency of the lowest band gap, equal to -31.51% and -31.02% for the isotactic and syndiotactic configurations, respectively.

This confirms that the magnitude of the frequency shift is not strongly geometry-dependent. Instead, the response is primarily governed by the viscoelastic properties of the constituent material and is general for PLA-based phononic structures.

S4 Generality and robustness of the predictive framework for additively manufactured polymer phononic materials.

S4.1 Robustness under elevated temperatures

The generality of the proposed framework in predicting the dynamic response of additively manufactured phononic structures under varying environmental conditions is assessed by analyzing its validity at elevated temperatures.

For polymer-based phononics, temperature plays a key role in governing viscoelastic behavior by influencing the effective stiffness and damping of structures. Since the framework relies on experimentally measured viscoelastic material properties, it requires these properties to be measured under non-ambient conditions. However, such characterization must be restricted to the glassy regime, where structural integrity is preserved. At temperatures approaching or exceeding the glass transition temperature, the material softens significantly, leading to a loss of structural shape and dimensional stability, rendering it unsuitable for transmission testing.

For PLA, the glass transition onset temperature is around 50 °C. To assess the validity of the proposed predictive framework at elevated temperatures, we characterized the material and conducted transmission tests (on the thin-ligament geometry shown in Fig. 2b) at 40 °C. Figure S13 illustrates the experimental setups used for mechanical characterization and transmission measurements under elevated-temperature conditions. In both setups, the samples are heated with flexible polyimide (PI) heating elements[4] that wrap around the specimens without physical contact. The heating elements were powered using a 3.7 V, 2.124 A supply, resulting in controlled heat generation. A small air gap (ranging from millimeters to centimeters) is maintained between the heating elements and the sample surface, such that heat transfer occurs primarily through convection. This configuration avoids direct thermal contact and hence results in no change to the boundary conditions. Using this approach, the samples reach an average temperature of approximately

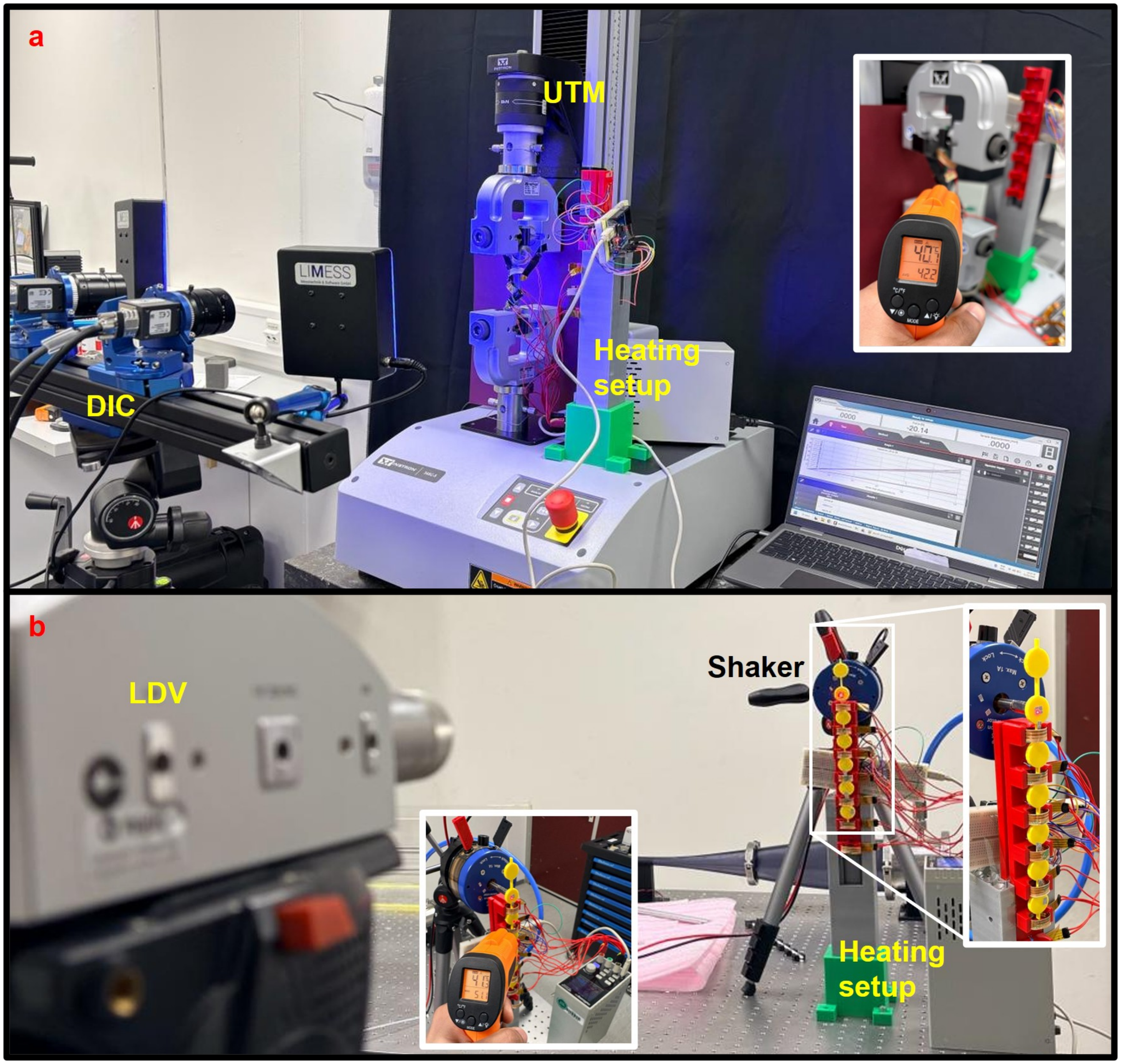

Fig. S13 (a) The experimental setup with the universal testing machine (UTM) and digital image correlation (DIC) to estimate Young's modulus and Poisson's ratio, respectively. (b) The setup for transmission measurements with a laser Doppler vibrometer (LDV). In both setups, the samples are heated by thermal chips wrapped around them; a magnified view is shown in (b). A heat gun is used to measure the sample temperature during testing, as shown in (a) and (b).

40 °C, as measured by a heat gun and indicated in the insets of Figs.S13a-b.

The measured material response is summarized in Figs. S14a-c. As shown, increasing temperature decreases the stiffness-related properties and leads to a moderate increase in damping. Specifically, the Young's modulus decreases by 5.7%, the Poisson's ratio by 1.8%, and the mean shear storage modulus by 7.9%, while the mean shear loss modulus increases by 5.6%, compared to ambient conditions (18 °C). These changes reflect the temperature-dependent viscoelastic behavior of PLA within the glassy regime. The influence of these modified properties on wave propagation is shown in Fig. S14d, where the transmission response exhibits a shift of the band-gap frequencies toward lower values. The central frequency of the Bragg band gap decreases by 3.7%, while its width is reduced by 12.8%. The close agreement between numerical predictions and experimental results demonstrates that the framework accurately captures the band-gap characteristics at the elevated temperature.

These results demonstrate that the proposed framework remains valid at elevated temperatures, provided that the corresponding mechanical properties are available. Since experimentally measured material parameters are required as inputs, the framework is

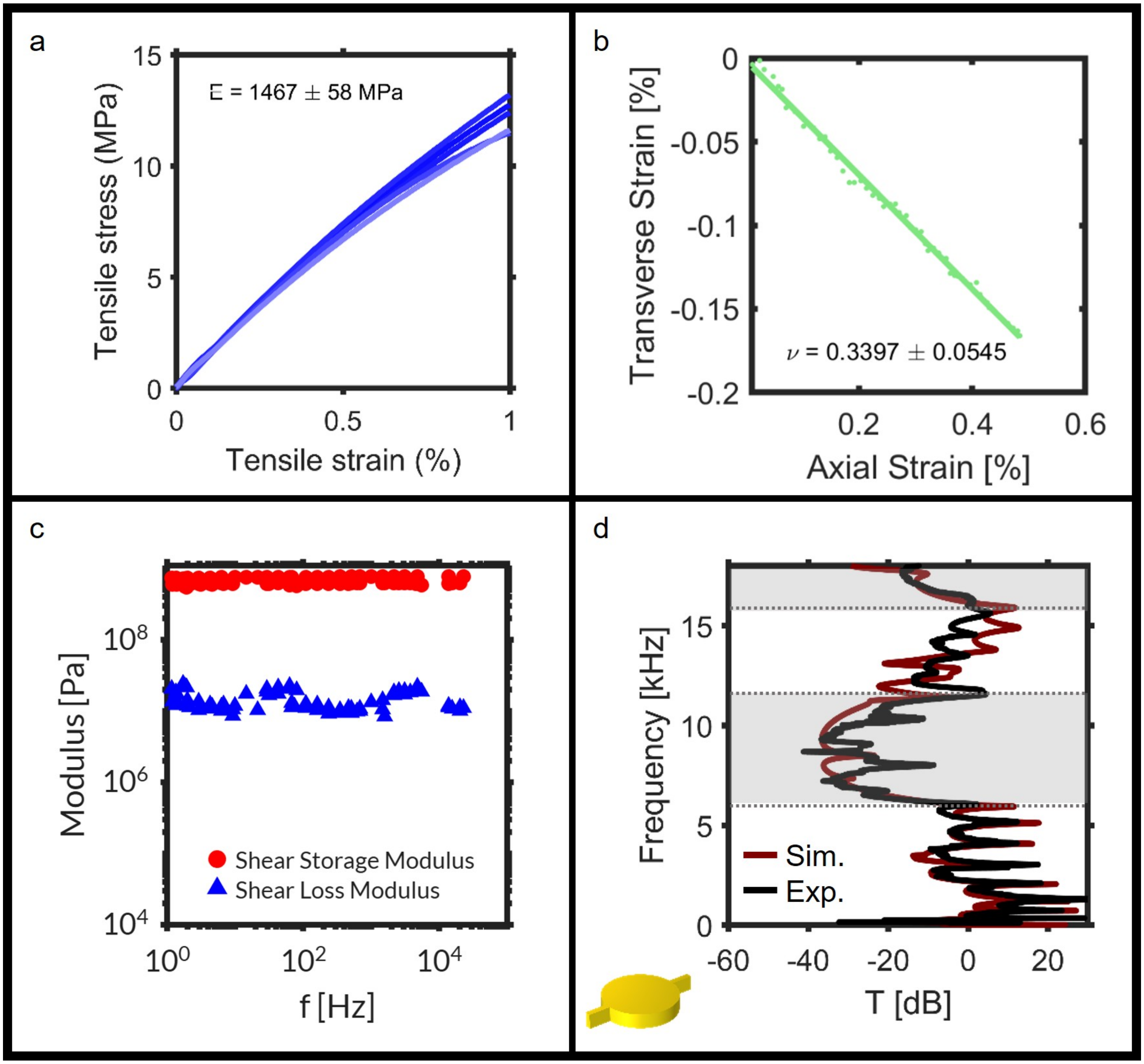

Fig. S14 (a) Stress–strain curves for a PLA dog-bone specimen at 40°C obtained from tensile tests following the ISO-527 standard. (b) Transverse strain versus axial strain measured using a DIC setup at 40°C to estimate Poisson's ratio. (c) DMA master curves shifted to the reference temperature of 40°C. (d) Comparison between numerical and experimental transmission results at 40°C. Numerically predicted band gaps are highlighted in grey, while the bounds of experimentally measured band gaps are indicated by dotted lines.

inherently flexible and can be readily extended to different environmental conditions, including varying humidity levels or lower temperatures, provided relevant mechanical properties are available. We emphasize, however, that the framework is primarily applicable within the glassy regime, where viscous losses remain moderate and structural integrity is preserved.

S4.2 Applicability to complex phononic architectures

To illustrate the applicability of our predictive framework to more complex phononic configurations than the disk-ligament structures analyzed in the main text, we consider here three-dimensional chiral chain structures previously reported in Ref.[3]. In the original study, they are presented in two forms, isotactic and syndiotactic, and fabricated by selective laser sintering (SLS) from a fiber-reinforced polyamide. Their numerical results and experimental measurements reveal notable discrepancies in band-gap position, width, and the frequencies of transmission peaks.

We fabricated the same structures from PLA on an FDM Bambu Lab X1C printer (see the insets in Figs. S15a-b), measured the wave transmission curves, and compared them with numerical predictions obtained using the proposed approach using the mechanical

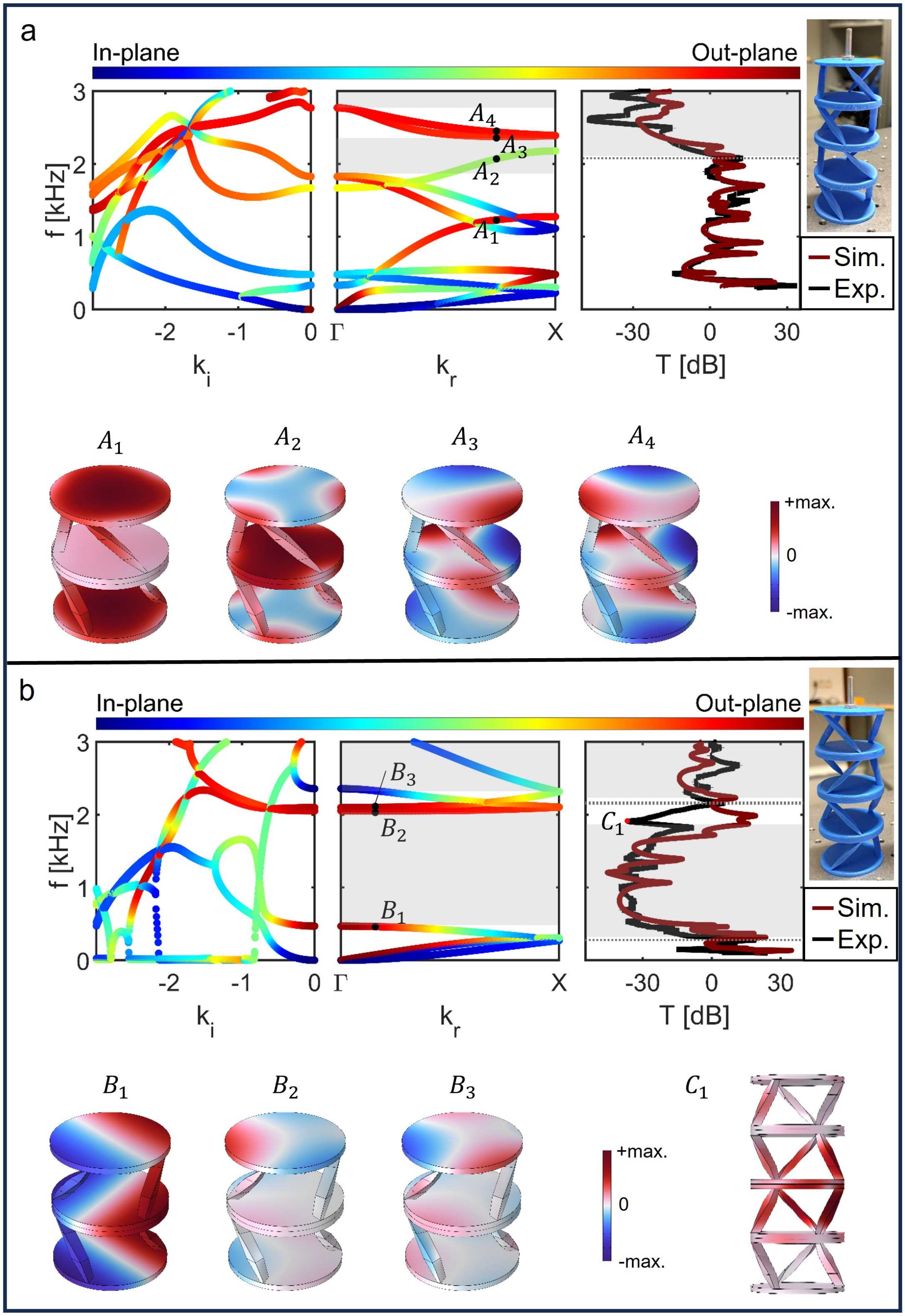

Fig. S15 Wave propagation characteristics of (a) isotactic and (b) syndiotactic phononic structures proposed in Ref.[3], including band structure diagrams, transmission curves, and vibration patterns at selected modes obtained numerically. The 3D-printed PLA samples are shown on the right, together with the glued screws used as connectors to a shaker. Numerically predicted band gaps are shaded in grey, and the bounds of the experimental band gaps are indicated by dotted lines.

properties of PLA from the main text. The predicted and measured responses exhibit excellent agreement in band-gap frequencies, transmission levels, and the overall spectral features for both structures, see Fig. S15. A minor deviation is observed in a single

transmission peak around 1.9 kHz in the syndiotactic chain (denoted as C_1), which is not experimentally excited because it is non-symmetric and strongly localized and therefore cannot be activated by the applied excitation at the edge.

The excellent agreement between our experimental and numerical data underscores the applicability and high accuracy of the proposed framework for three-dimensional phononic structures (i.e., beyond plate-type designs) and demonstrates that the long-standing mismatch between numerical predictions and experiments can be substantially reduced by incorporating experimentally measured material properties of 3D-printed constituents.

S4.3 Applicability to different additive manufacturing techniques

To assess the validity of the proposed framework to phononic configurations produced by other additive manufacturing techniques, we manufactured the straight thin–ligament designs from a hard photopolymer resin (ABS-like 3.0, Elegoo) by stereolithography (SLA) using a Uniformation GK3 Ultra printer. After fabrication, the samples were post-processed with a 5-minute thermal treatment, followed by a 5-minute UV cure.

The quasi-static Young's modulus and Poisson's ratio, together with the frequency-dependent storage and loss moduli and temperature-dependent characteristics obtained experimentally, are presented in Figs. S16a-e. The density of the SLA samples is $1252.3\,\mathrm{kg\,m^{-3}}$.

Figure S16f shows the corresponding dispersion and transmission characteristics of the straight thin-ligament phononic structure (Fig. 2b), obtained using the proposed framework, alongside the experimental transmission response. The discrepancies between numerical predictions and experimental measurements are quantified in Table S3 and are found to be comparable to those observed for the FDM-fabricated structures (Table S2). This allows us to conclude that the framework can be used to predict the wave dynamics in phononic structures produced by a broader range of additive manufacturing techniques, not only FDM.

Also, given that SLA guarantees at least one order higher printing resolution than FDM, with negligible void content and improved interlayer bonding, the similar level of agreement between the two cases indicates that manufacture-induced defects in FDM structures have a limited impact on the predicted wave response. This further supports the conclusion that the predictive accuracy of the framework is primarily governed by accurate material characterization rather than by the specific additive manufacturing technique employed.

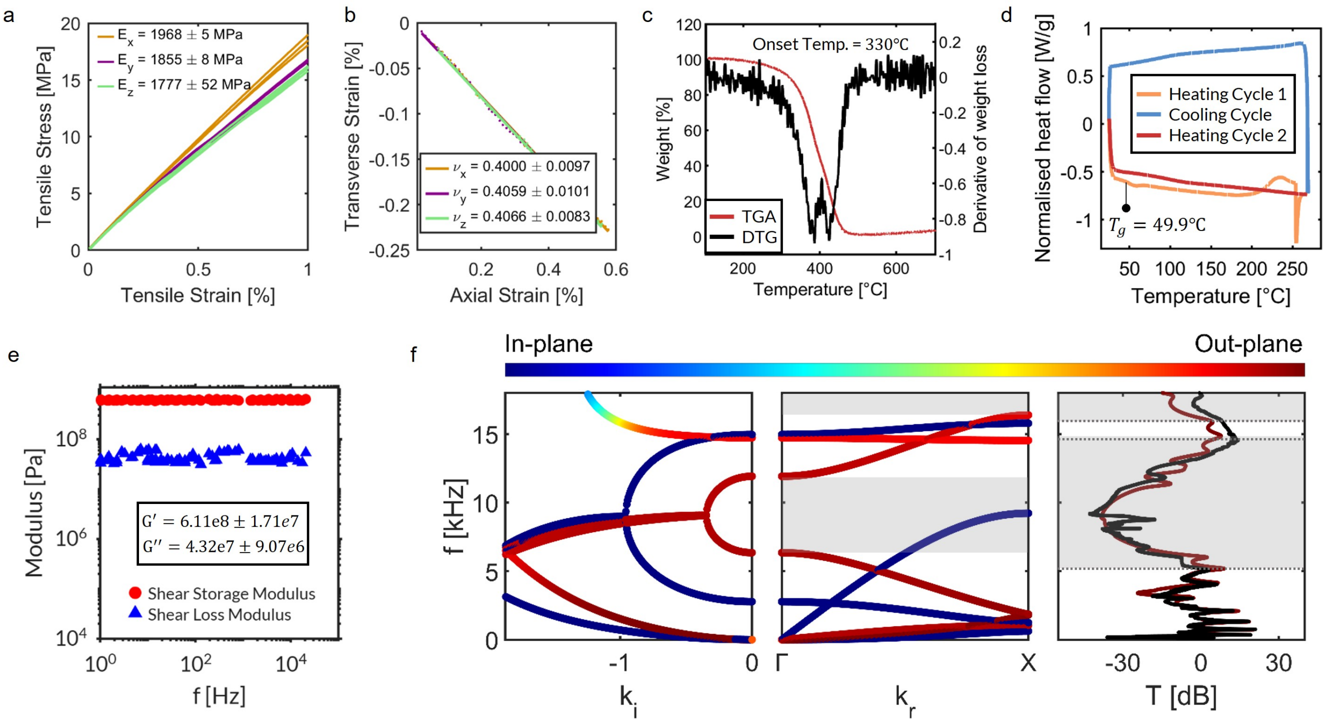

Fig. S16 Material characterization and wave propagation analysis for SLA-printed samples. (a) Stress–strain curves for samples printed in three orientations: flat (x), on-edge (y), and upright (z); the corresponding Young's modulus values are indicated in the legend. (b) Poisson's ratio for the same configurations. (c) Thermogravimetric analysis (TGA) results for the SLA-printed material. (d) Differential scanning calorimetry (DSC) results, where the first heating cycle indicates a glass transition temperature of approximately $49.9\,^{\circ}\mathrm{C}$. (e) DMA master curves shifted to a reference temperature of $18\,^{\circ}\mathrm{C}$, showing the frequency-dependent variation of shear storage and shear loss moduli. (f) Real and imaginary band structure diagrams together with a comparison between numerical and experimental transmission responses. Numerically predicted band gaps are shaded in grey, and the bounds of experimentally observed band gaps are marked by dotted lines.

Table S3 Quantitative comparison between the experimental results and numerical predictions for the straight thin-ligament geometry (Fig. 2b) printed on SLA printer, including band-gap mid-frequency, band-gap width, and attenuation depth, along with their respective deviations from experimental values.

Case	Band-gap position			Band-gap characteristics			
	Mid-freq [kHz]	Deviation [Hz]	Deviation [%]	BG width [kHz]	BG width [%]	Attenuation depth [dB]	Deviation [%]
Experimental	9.82	0	0	9.51	100	−50.8	0
Numerical	10	−175.6	−1.8	9.66	101.6	−47.3	6.9

References

1 Bambu Lab, *Bambu PLA Basic Technical Data Sheet*, https://files.bbystatic.com/G8TU2klvsR6MVeDE4zgPkw%3D%3D/Bambu%2BFilament%2B-Technical%2BData%2BSheet, 2023, Accessed: 02 Jun 2026.

2 Bambu Lab, *Bambu ABS Technical Data Sheet*, https://store.bblcdn.com/s7/default/23b4cf2b83d5470bb96d19970b5f3ae8/Bambu_ABS_Technical_Data_Sheet_V3.pdf, 2023, Accessed: 02 Jun 2026.

3 A. Bergamini, M. Miniaci, T. Delpero, D. Tallarico, B. Van Damme, G. Hannema, I. Leibacher and A. Zemp, *Nature communications*, 2019, **10**, 4525.

4 Sourcing Map, *Polyimide Heating Film, 12 V, 12 W, 93 mm × 10 mm*, https://www.amazon.nl/sourcing-Verwarmingsfolie-verwarmingsplaat-verwarmingselementen-verwarmingsstrips/dp/B0CTT46BKY, n.d., Accessed: 02 Jun 2026.
